# Supplementary material for: Contrasting temporal dynamics of land surface temperature responses to different types of forest loss
Source: Innovation (Camb). 2025 Mar 11;6(6):100875. doi: 10.1016/j.xinn.2025.100875 (PMC12169273; doi:10.1016/j.xinn.2025.100875)
Supplement: Document S1. Figures S1–S17 and Table S1 [file mmc1.pdf]

The Innovation, Volume *6*

Supplemental Information

Contrasting temporal dynamics of land surface temperature responses to different types of forest loss

Jing Li, Zhao-Liang Li, Xiangyang Liu, Yitao Li, Meng Liu, Nanshan You, Hua Wu, Lei He, Menglin Si, Ronglin Tang, Chenghu Zhou, Wei Zhao, Si-Bo Duan, Pei Leng, Wenqi Liu, Enyu Zhao, Bo-Hui Tang, and Zhenong Jin

This PDF file includes:

- Supplementary Text
- Supplementary Figures (Figures S1-17)
- Supplementary Tables (Table S1)

Supplementary Text

Advantages of 10-day time-series data and the effectiveness of the improved BEAST method in data loss scenarios

Figure S5 shows the change detection results using the improved BEAST method at a forest loss sample site in India, where forest loss occurred in 2008 (Figure S5a). By employing high temporal resolution LST time series, the method successfully captured the abrupt LST changes caused by the land use transition from forest to cropland and revealed the distinct trend trajectories: a warming effect following loss and the cooling effect due to afforestation, as planted forests covered the loss pixel in 2018 (Figure S5b). In contrast, the annual mean time series underestimated the LST trend due to mid-year data loss (Figure S5c). Moreover, our results generalize LST trends into key features, smoothing out annual data fluctuations and enabling users to focus on the primary gradual and abrupt changes in LST. Figure S6 provides another example of forest loss driven by commodity production in the Amazon (Figure S6a). Our results captured the warming effect of crop expansion and demonstrated that trends detected using high temporal resolution data effectively avoid overestimation caused by missing data during the rainy season (Figures S6b-c). Notably, the seasonal component proved highly sensitive to forest loss. As early as 2003, when land cover within the loss pixels began transitioning (partially changing from forest to cropland), an abrupt change was detected in the seasonal component. Results from a forest loss sample driven by fire (Figure S7) further confirmed the superiority of high temporal resolution time series in characterizing abrupt and gradual LST changes compared to annual data.

Similarly, Figure S11 shows the change detection results for albedo at the three selected sample sites. The improved BEAST effectively captured the abrupt increase in albedo associated with the transition from forest to cropland at sites 1 and 2 (Figures S11a-b), despite significant data loss at these locations (Figures S11d-e). Additionally, our findings demonstrate the method's robustness in the presence of outliers, such as those observed in 2001 and 2009 at site 3. The method successfully detected a decrease in albedo following a fire, attributed to the deposition of black carbon on soil surfaces and dead trees.

Supplementary Figures

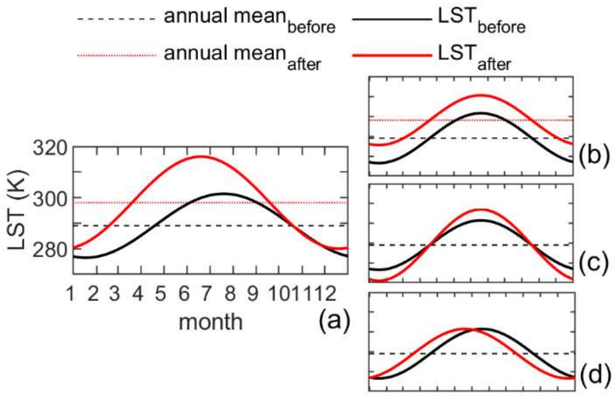

Figure S1. Framework for increase in monthly LST time series. The whole season warming (a) is driven by three distinct factors: (b) an increase in the annual mean LST, (c) an amplified amplitude of the seasonal cycle, and (d) an earlier phase shift in the seasonal cycle. Black lines represent LST time series before the change, red lines represent time series after the change. Dash or dot lines represent annual mean values.

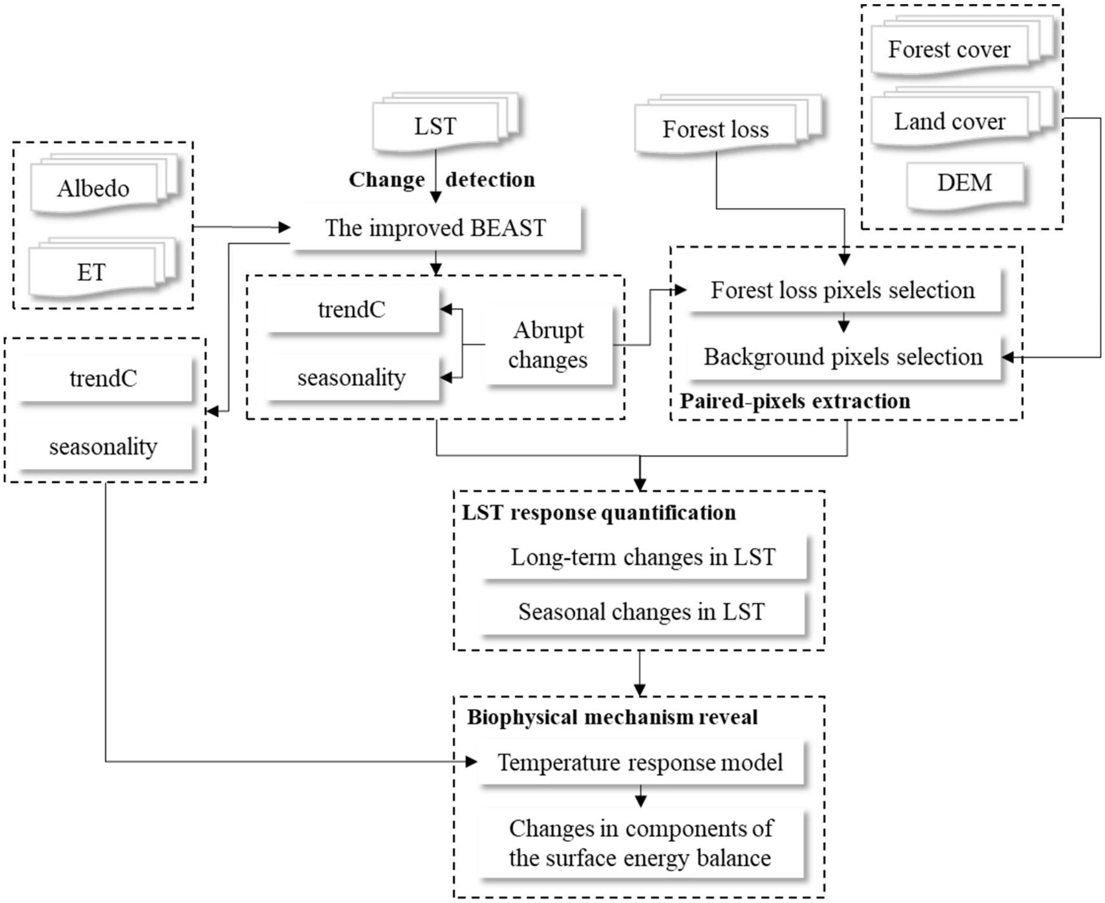

Figure S2. Flowchart of the assessment of biophysical impacts of forest loss based on satellite observations.

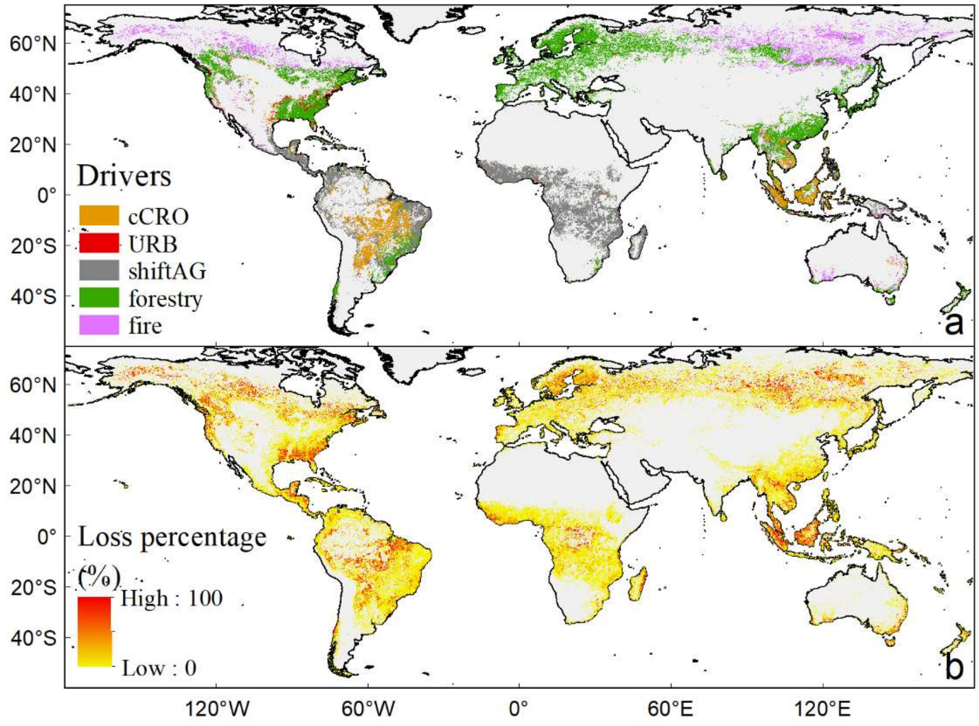

Figure S3. Spatial distribution of different forest loss types (a) and its loss percentage (b). Forest loss is primarily driven by five factors: commodity driven conversion from forest to cropland (cCRO), Urbanization (URB), shifting agriculture (shiftAG), forestry, and fire. The Grid cells with a spatial resolution of 1 km.

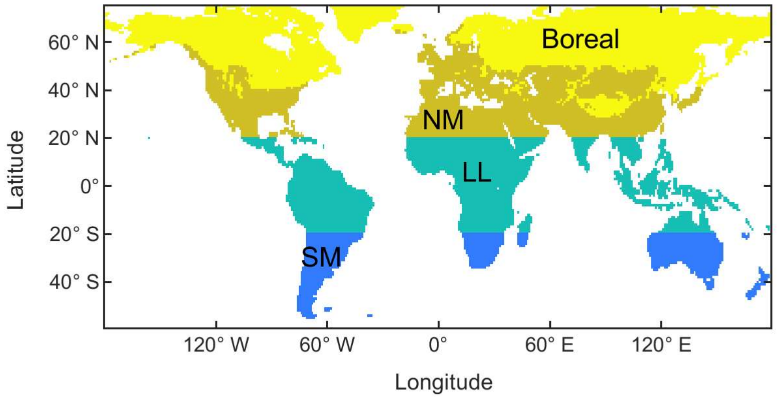

Figure S4. The distribution of different climate zones: southern mid-latitudes (SM), low latitudes (LL), northern mid-latitudes (NM), and boreal zones. The SM are the area between 60° S and 20° S, the LL are the area between 20° S and 20° N, the boreal region is the boreal zone defined on the Köppen–Geiger world map above 20° N, and the NM are the area that the left area above 20° N.

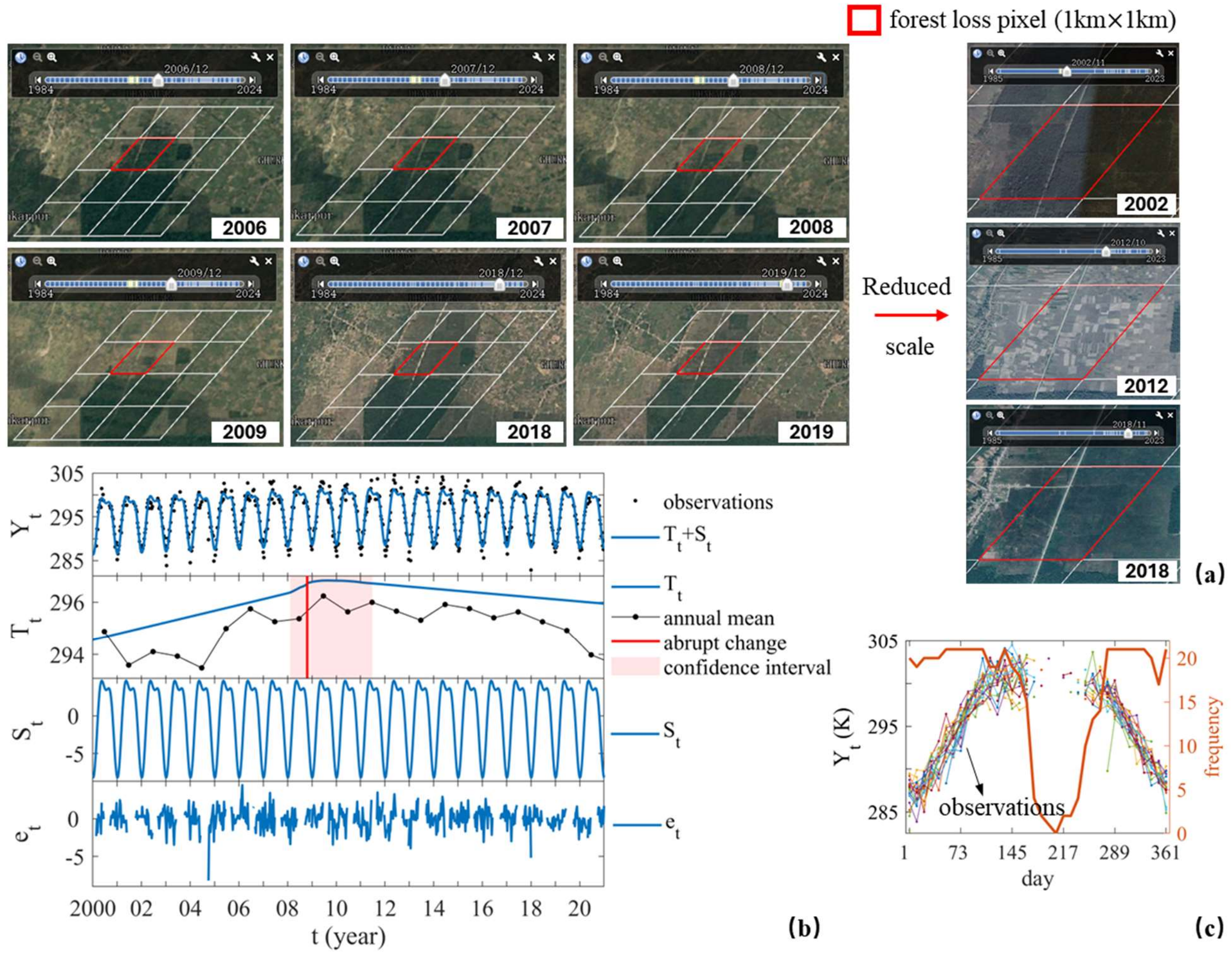

Figure S5. Forest loss sample driven by commodity production and its change detection results for LST. (a) High spatial resolution image sequences from Google Earth highlight a crop expansion after 2008 followed by afforestation in India. MODIS pixels are shown in white boxes, and the forest loss pixel is shown in the red box (central at 80.03 °E, 20.25 °N). (b) Change detection results for LST by using the improved BEAST. The LST observations with a temporal resolution of 10-day are displayed as black dots. The decomposed trend (T_t), seasonal (S_t), and remainder (e_t) components are displayed as blue lines. The annual mean LST is displayed as black line with dots, and the detected abrupt changes and their confidence intervals are shown as red lines and light red fill. (c) The observed LST time series and their frequency in the intra annual scale. The 'day' in x-axis label refers to the specific day of the year.

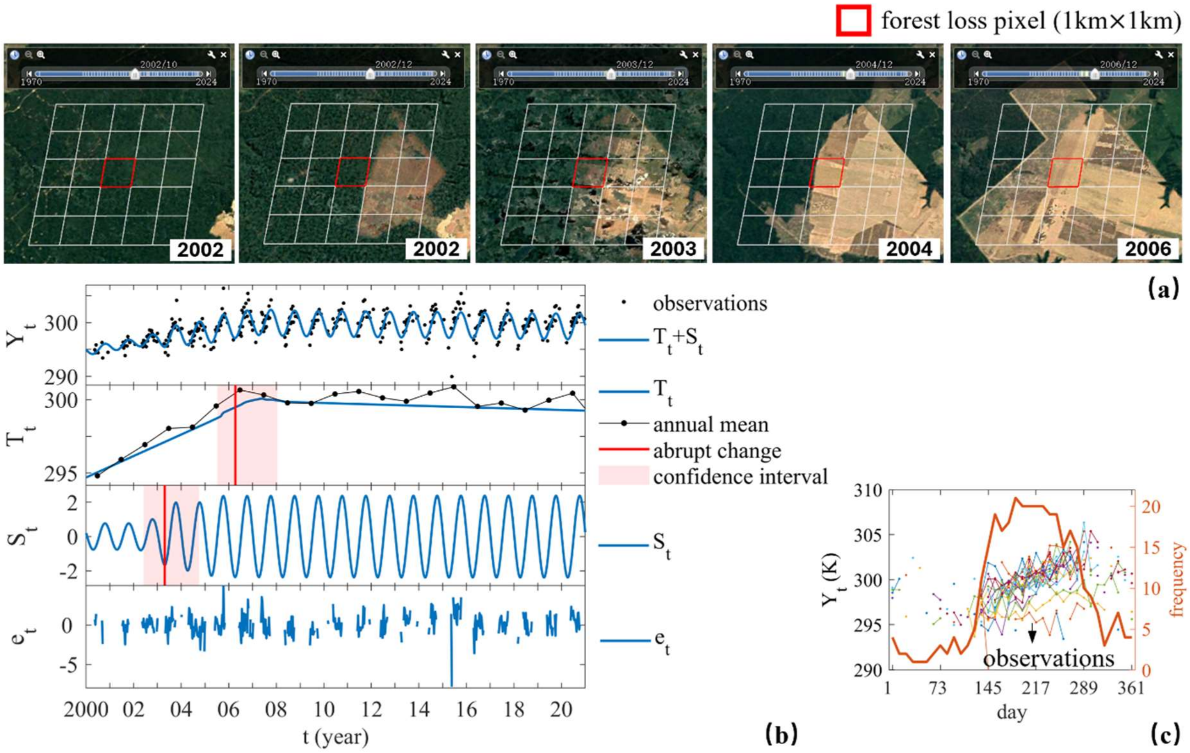

Figure S6. Same as Figure S5 but for a forest loss sample driven by commodity after 2003 in the Brazilian Amazon (central at 47.87 °W, 3.87 °S).

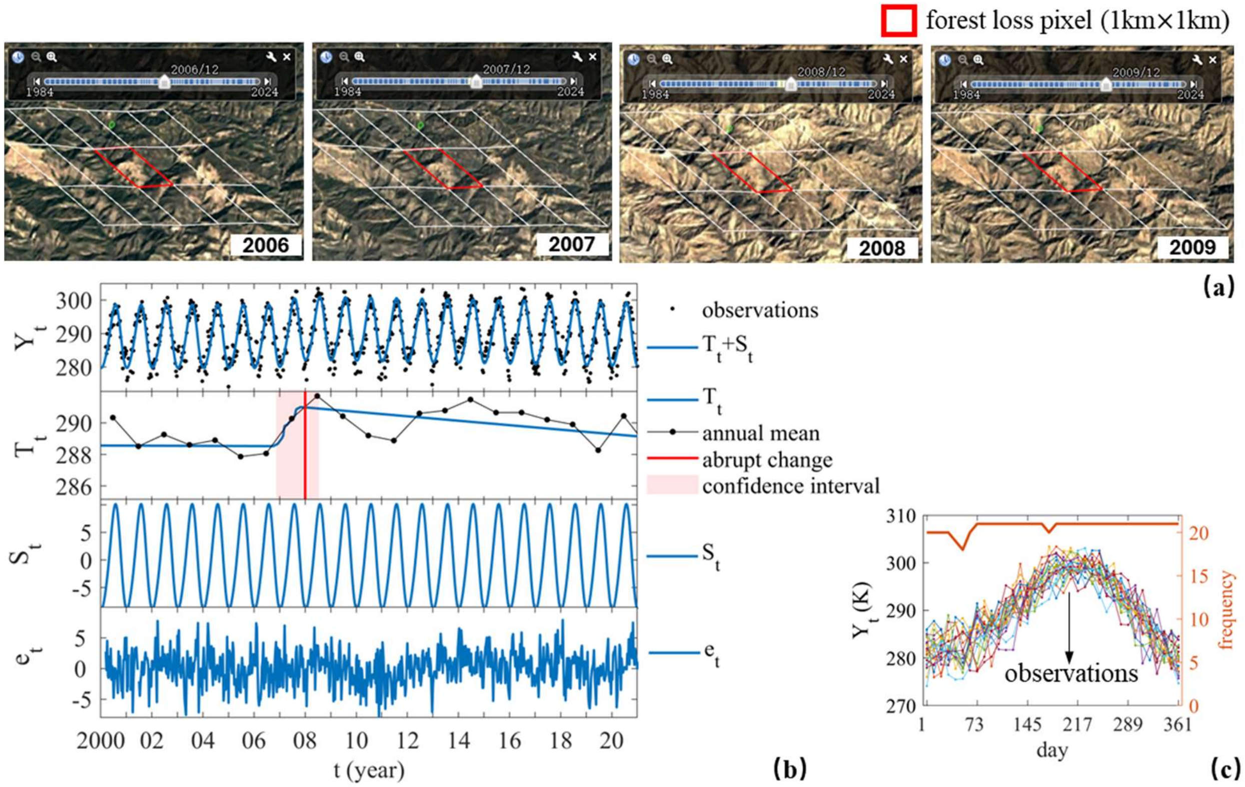

Figure S7. Same as Figure S5 but for a forest loss sample driven by wildfire that occurred in California, the USA in 2008 (central at 119.59 °W, 34.64 °N).

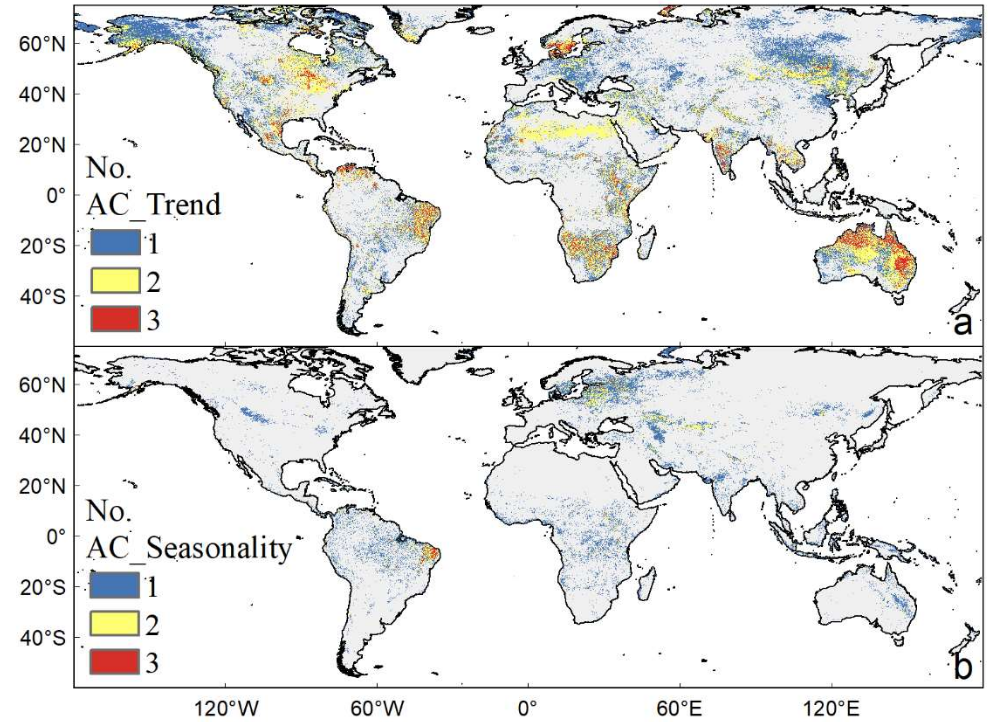

Figure S8. Abrupt changes (AC) in time-series LST. The numbers of abrupt changes in trend (a) and seasonal (b) components detected by the improved BEAST by using the MODIS LST time series. The Grid cells with a spatial resolution of 1km.

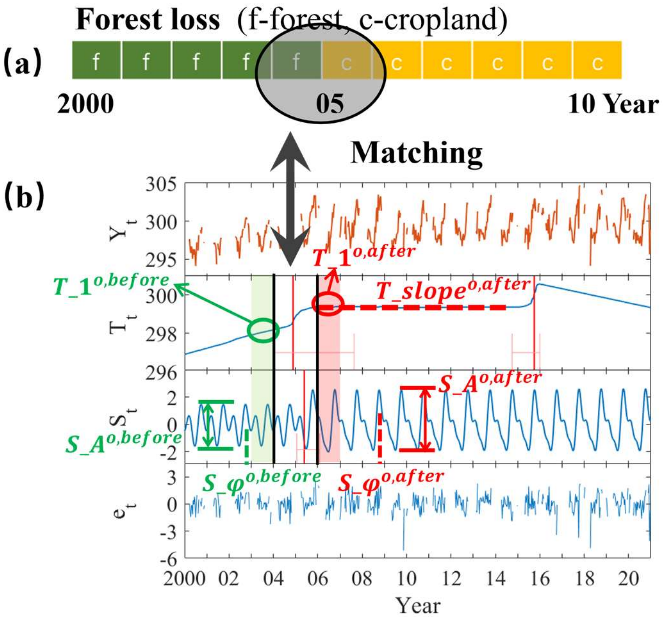

Figure S9. Illustration of the matching process between (a) a known forest loss event and (b) the corresponding detected abrupt changes in LST. (b) is the same as the Figure S10 but for commodity-driven forest loss in 2005 in the Brazilian Amazon. The calculation of the changes in LST trend and seasonality for a forest loss pixel are shown.

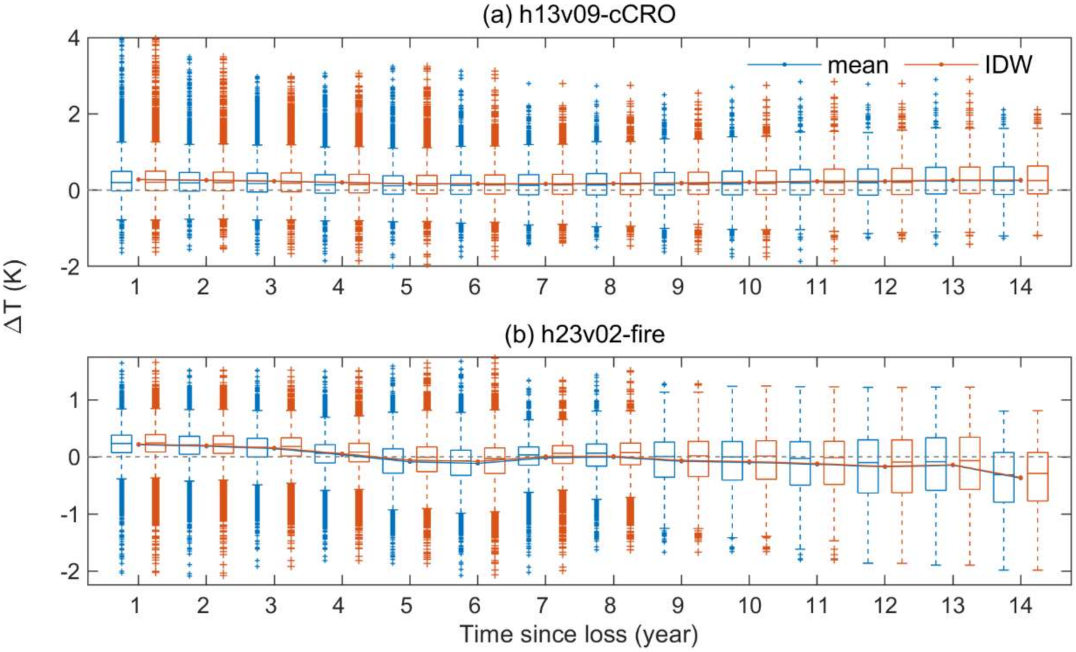

Figure S10. **Temporal dynamics of Land Surface Temperature (LST) trendC (ΔT) from 1 year to 14 years after the forest loss at two selected MODIS tiles: h13v09 and h23v02.** The primary causes of forest loss were cropland conversion (cCRO) (a) and fire (b), respectively. The ΔT dynamics was calculated by employing two different methods for extracting background pixel signals: the mean method (blue boxes) and the Inverse Distance Weighting (IDW) method (red boxes). The lines indicate the mean values of ΔT derived from the two extraction methods (mean and IDW).

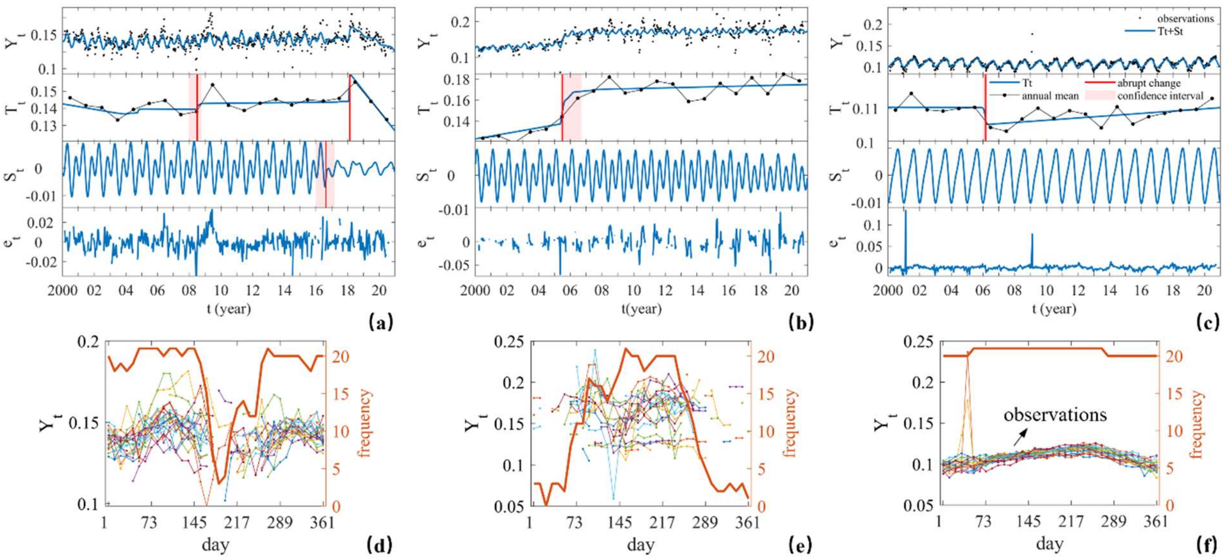

Figure S11. Same as Figure S10 but for change detection results for albedo.

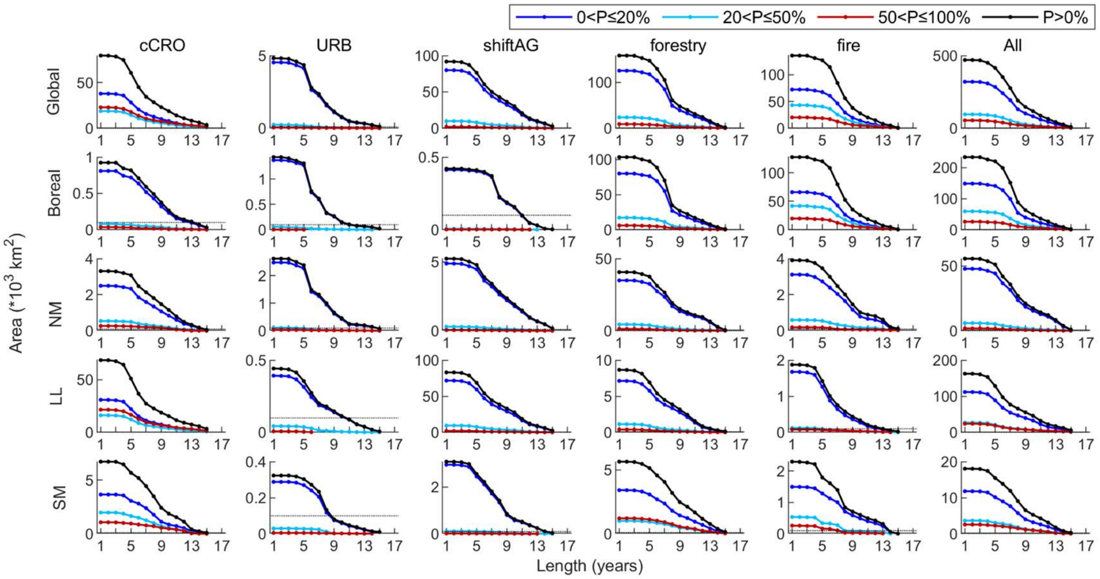

Figure S12. The area of extracted forest loss event driven by cCRO, URB, shiftAG, forestry, fire, and all types of forest loss across global, boreal, NM, LL, and SM zones. P is the percentage of forest loss.

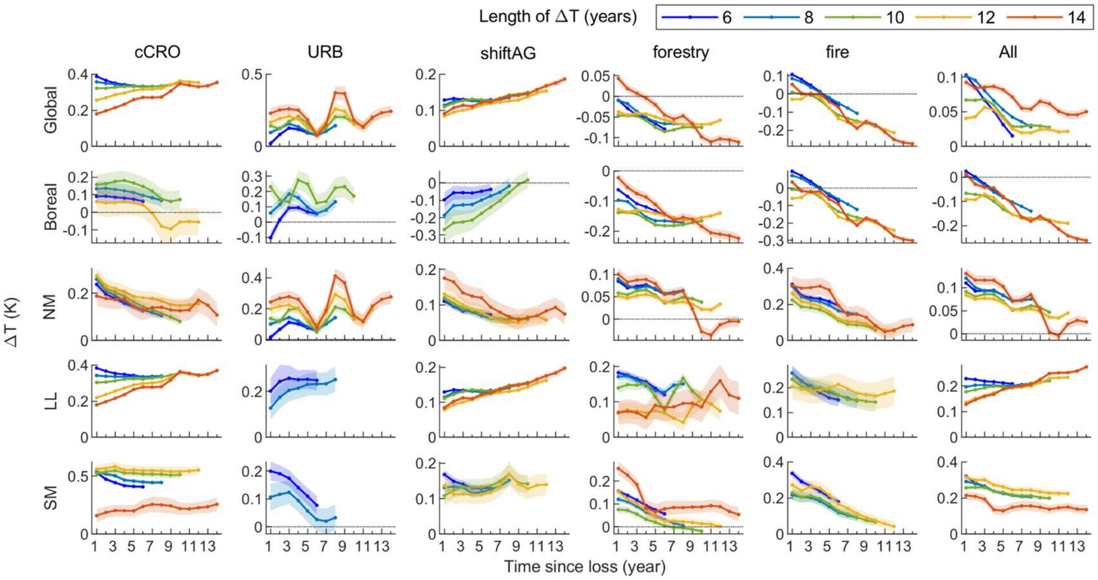

Figure S13. Temporal dynamics of the ΔT from one year to 14 years after the forest loss driven by cCRO, URB, shiftAG, forestry, fire, and all types of forest loss across global, boreal, NM, LL, and SM zones. Lines of different colors represent the different lengths (6 to 14 years) of ΔT. The filled area represents 95% confidence intervals.

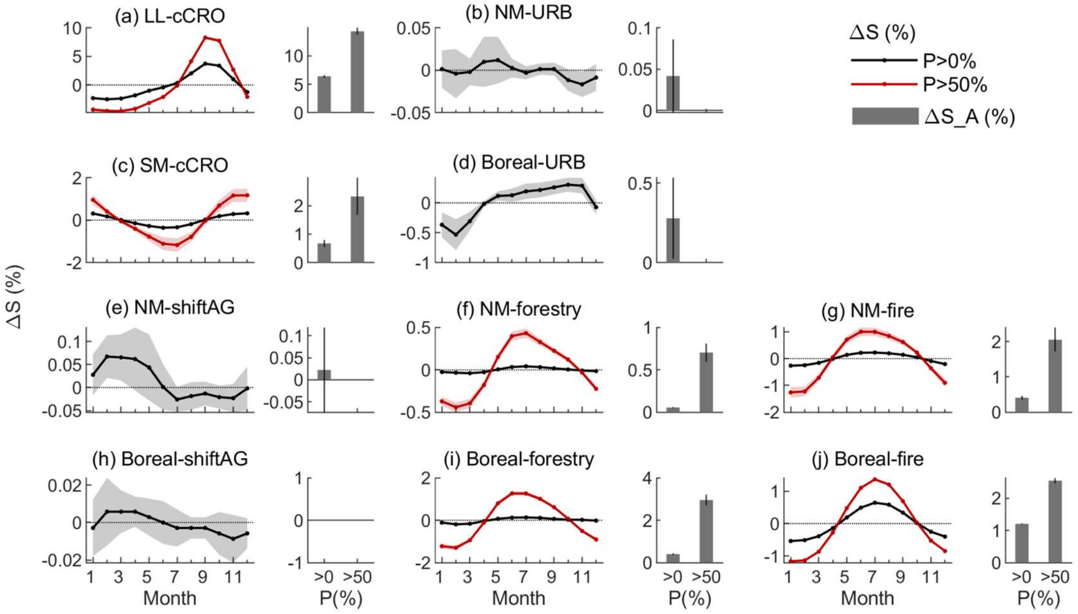

Figure S14. Relative changes in seasonal component in different months (ΔS) and seasonal amplitude (ΔS_A) caused by the forest loss driven by cCRO, URB, shiftAG, forestry, and fire across different climate zones. Subfigure titles indicate 'climate zone-forest loss type'. Colored lines on the left side represent ΔS with loss percentage (P) greater than 0% (black) and 50% (red), respectively. The filled area represents 95% confidence intervals.

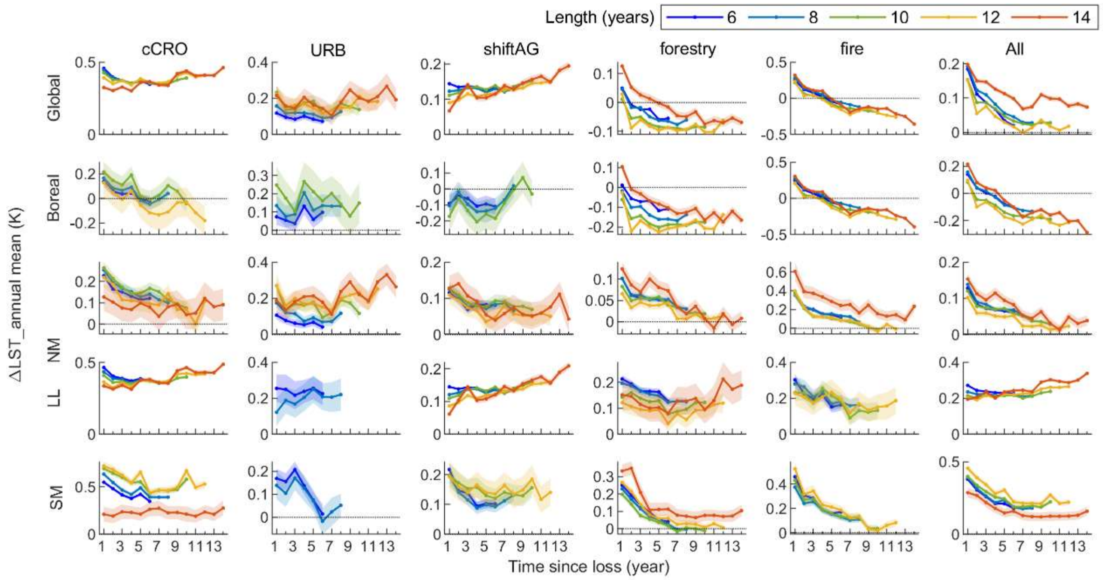

Figure S15. Temporal dynamics of the $\Delta LST\_annual\ mean$ from one year to 14 years after the forest loss driven by cCRO, URB, shiftAG, forestry, fire, and all types of forest loss across Global, Boreal, NM, LL, and SM boreal zones. Lines of different colors represent the different lengths of $\Delta LST\_annual\ mean$. The filled area represents 95% confidence intervals.

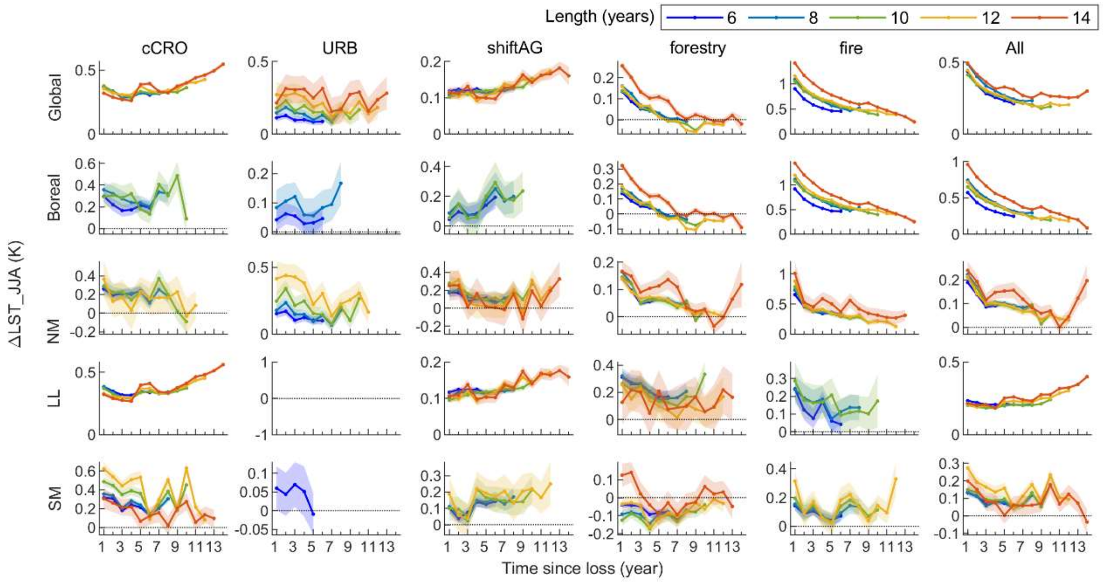

Figure S16. Same as FigureS15 but for ΔLST_JJA. JJA represents June-July-August.

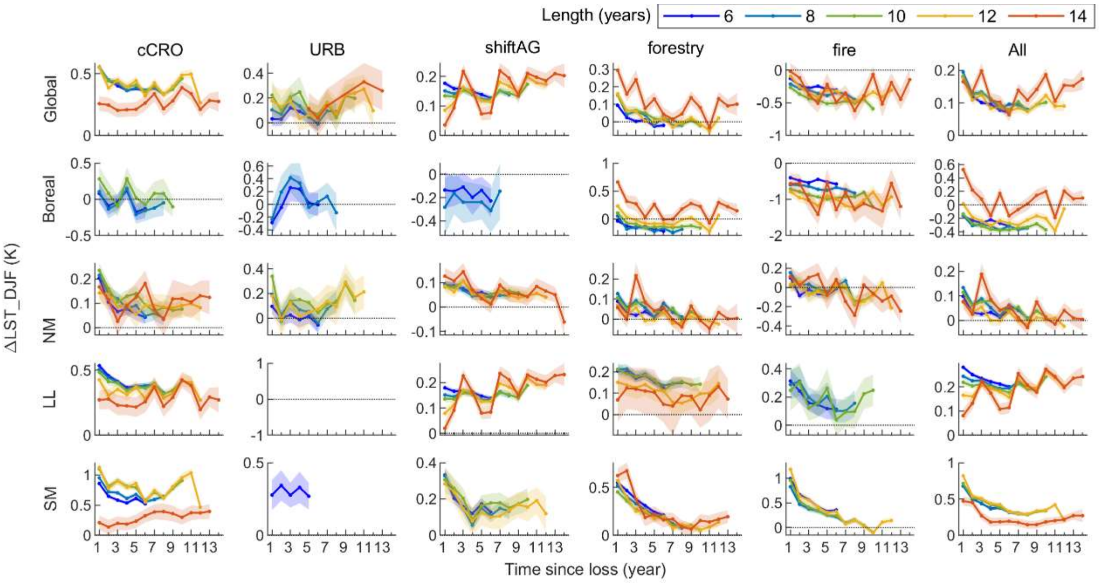

Figure S17. Same as Figure S15 but for ΔLST_DJF. DJF represents December-January-Feburary.

Supplementary Tables

Table S1. Area percentages of different change patterns of LST trendC following forest loss across different climate zones.

Abrupt impact	Long-term impact following abrupt impact	Climate zones				
		SM (%)	LL (%)	NM (%)	Boreal (%)	Global (%)
warming	enhanced warming (+ +)	0.6	6.4	1.6	2.8	11.4
	abrupt warming (+ ~)	0.9	10.1	4.8	6.1	21.9
	attenuated warming (+ -)	1.3	9.5	4.9	13.1	28.8
cooling	attenuated cooling (- +)	0.4	3.7	2.7	3.5	10.4
	abrupt cooling (- ~)	0.4	4.2	4.6	5.5	14.8
	enhanced cooling (- -)	0.3	1.9	2.1	7.9	12.2
insignificant change (~)		0.0	0.1	0.1	0.2	0.4
sum		4.0	36.0	20.8	39.1	100.0
