# Supplementary material for: Contrasting temporal dynamics of land surface temperature responses to different types of forest loss
Source: Innovation (Camb). 2025 Mar 11;6(6):100875. doi: 10.1016/j.xinn.2025.100875 (PMC12169273; doi:10.1016/j.xinn.2025.100875)
Supplement: Document S2. Article plus supplemental information [file mmc2.pdf]

ARTICLE

OPEN ACCESS

Contrasting temporal dynamics of land surface temperature responses to different types of forest loss

Jing Li,[1] Zhao-Liang Li,[1,2,*] Xiangyang Liu,[1] Yitao Li,[2] Meng Liu,[1] Nanshan You,[3] Hua Wu,[4] Lei He,[1] Menglin Si,[1,2] Ronglin Tang,[2] Chenghu Zhou,[5] Wei Zhao,[6] Si-Bo Duan,[1] Pei Leng,[1] Wenqi Liu,[7] Enyu Zhao,[8] Bo-Hui Tang,[9] and Zhenong Jin[10,11,12]
*Correspondence: lizhaoliang@caas.cn

Received: September 21, 2024; Accepted: March 7, 2025; Published Online: March 11, 2025; https://doi.org/10.1016/j.xinn.2025.100875

© 2025 The Author(s). Published by Elsevier Inc. on behalf of Youth Innovation Co., Ltd. This is an open access article under the CC BY license (http://creativecommons.org/licenses/by/4.0/).

GRAPHICAL ABSTRACT

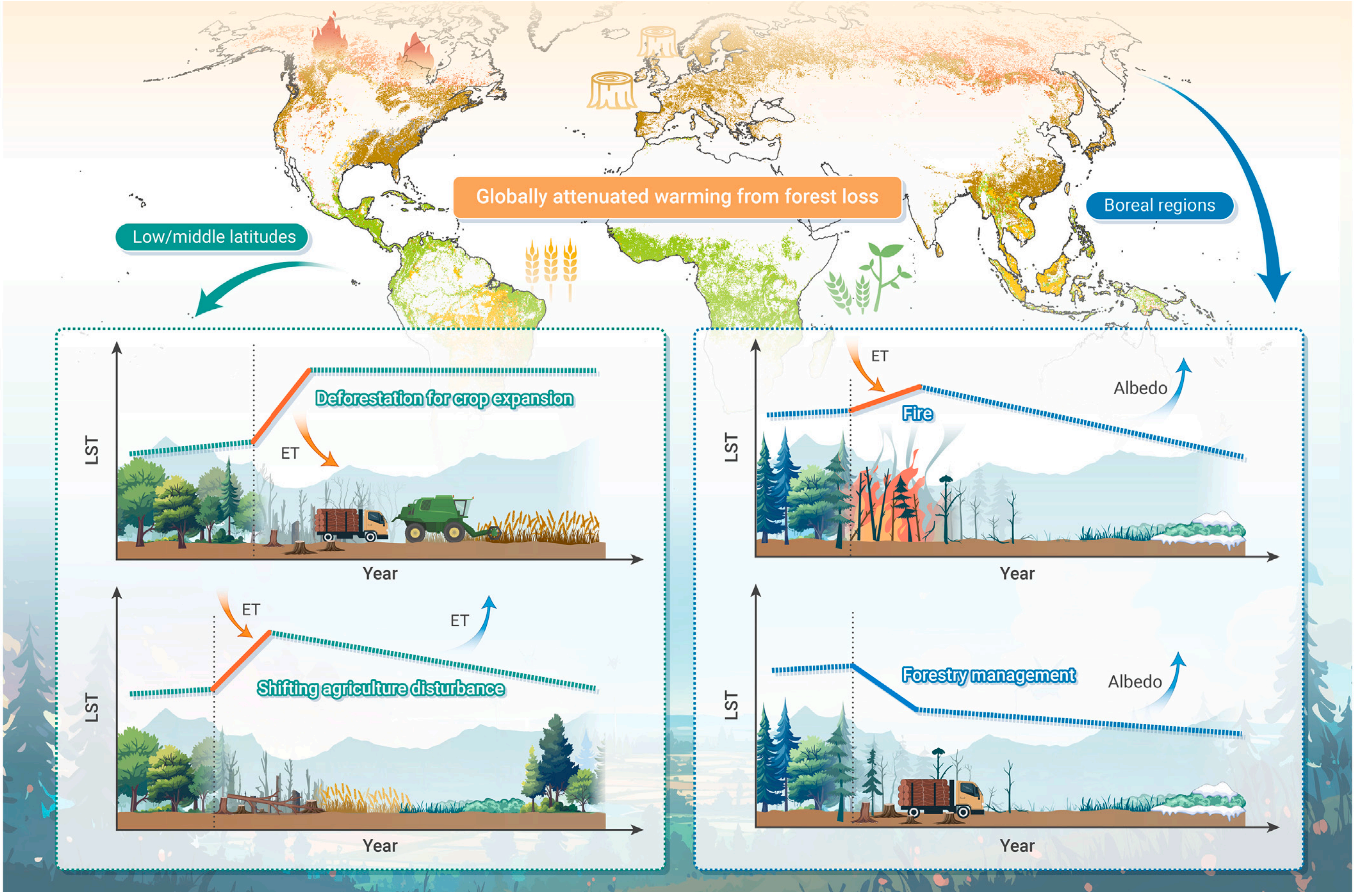

PUBLIC SUMMARY

- The impact of forest loss on land surface temperature (LST) can persist, intensify, or weaken over time.
- LST rises by a global average of 0.12 K one year after forest loss, but recovered by −0.14 K per decade.
- Warming persists or weakens in low and mid-latitudes, while cooling intensifies in boreal regions.
- Temporal dynamics of LST response to forest loss should be considered in climate feedback assessments.

Check for updates
OPEN ACCESS

ARTICLE

The Innovation

Contrasting temporal dynamics of land surface temperature responses to different types of forest loss

Jing Li,[1] Zhao-Liang Li,[1,2,*] Xiangyang Liu,[1] Yitao Li,[2] Meng Liu,[1] Nanshan You,[3] Hua Wu,[4] Lei He,[1] Menglin Si,[1,2] Ronglin Tang,[2] Chenghu Zhou,[5] Wei Zhao,[6] Si-Bo Duan,[1] Pei Leng,[1] Wenqi Liu,[7] Enyu Zhao,[8] Bo-Hui Tang,[9] and Zhenong Jin[10,11,12]

[1]State Key Laboratory of Efficient Utilization of Arable Land in China, Institute of Agricultural Resources and Regional Planning, Chinese Academy of Agricultural Sciences, Beijing 100081, China

[2]State Key Laboratory of Resources and Environmental Information System, Institute of Geographic Sciences and Natural Resources Research, Chinese Academy of Sciences, Beijing 100101, China

[3]Key Lab for Resources Use and Environmental Remediation, Institute of Geographic Sciences and Natural Resources Research, Chinese Academy of Sciences, Beijing 100101, China

[4]School of Resources and Environment, University of Electronic Science and Technology of China, Chengdu 610054, China

[5]Center for Ocean Remote Sensing of Southern Marine Science and Engineering Guangdong Laboratory (Guangzhou), Guangzhou Institute of Geography, Guangdong Academy of Sciences, Guangzhou 510070, China

[6]Institute of Mountain Hazards and Environment, Chinese Academy of Sciences, Chengdu 610041, China

[7]Department of Geography, Oklahoma State University, Stillwater, OK 74078, USA

[8]College of Information Science and Technology, Dalian Maritime University, Dalian 116026, China

[9]Faculty of Land Resource Engineering, Kunming University of Science and Technology, Kunming 650093, China

[10]Department of Bioproducts and Biosystems Engineering, University of Minnesota, St. Paul, MN 55108, USA

[11]Institute of Ecology, College of Urban and Environmental Science, Peking University, Beijing 100871, China

[12]State Key Laboratory for Vegetation Structure, Function and Construction (VegLab), Peking University, Beijing 100871, China

*Correspondence: lizhaoliang@caas.cn

Received: September 21, 2024; Accepted: March 7, 2025; Published Online: March 11, 2025; https://doi.org/10.1016/j.xinn.2025.100875

© 2025 The Author(s). Published by Elsevier Inc. on behalf of Youth Innovation Co., Ltd. This is an open access article under the CC BY license (http://creativecommons.org/licenses/by/4.0/).

Citation: Li J., Li Z.-L., Liu X., et al., (2025). Contrasting temporal dynamics of land surface temperature responses to different types of forest loss. The Innovation **6(6)**, 100875.

Forest loss impacts local climate through biophysical processes. However, our understanding of this impact remains limited due to the neglect of its temporal dynamics. Using a space-and-time scheme that incorporates a change-detection method, we assess the dynamics of land surface temperature (LST) responses to various forest-loss types. Globally, LST increased by 0.12 K one year after forest loss, followed by a decreasing trend of −0.14 K per decade. Deforestation driven by commodity production and urbanization results in persistent warming, while forest disturbances such as shifting agriculture, forestry, and fire trigger diverse response dynamics with significant spatial variation due to differences in subsequent vegetation recovery. These disturbances cause attenuated warming in low and mid-latitudes, while, in the boreal zone, contrasting dynamics are observed: shifting agriculture causes attenuated cooling, whereas forestry and fire result in enhanced cooling. In addition to amplifying the amplitude of the LST seasonal cycle, forest loss also shifts the seasonal phase, which has not been previously reported. These findings demonstrate that climate feedback from forest loss is climate specific, loss-type dependent, and time varying, providing new insights for the development of local climate policies.

INTRODUCTION

Forests worldwide are undergoing drastic changes, as exemplified by the loss of 4.7 million hectares of global forest from 2000 to 2020 due to deforestation, fire, and forestry management.[1,2] Forest loss affects land surface temperature (LST) through biophysical effects, arising from changes in radiative flux and non-radiative processes between the atmosphere and the land surface. These changes are driven by alterations in surface properties, such as surface albedo and roughness.[3–5] Generally, boreal forest loss is associated with LST decrease due to the enhanced reflection of shortwave radiation, while tropical forest loss tends to increase LST because of reduced evapotranspiration.[6,7] Knowledge of the climate effect of forest loss and its underlying biophysical mechanism is vital for mitigating adverse climate impacts through the development of targeted interventions.[8–10]

Forest loss alters LST by modifying both its trend and seasonality, as illustrated in Figure 1. It induces abrupt changes in LST trend components (hereafter abbreviated as trendC) due to rapid alterations in land surface properties.[11,12] It also leads to gradual changes in trendC, with post-loss trend trajectories diverging from pre-loss trajectories. These gradual changes are driven by subsequent land succession, such as vegetation recovery after wildfires and forestry management, cultivated land management, or intensive urban use after deforestation.[13,14] The dynamic response of LST to forest loss, characterized by abrupt and gradual changes in trendC, helps differentiate between the immediate impacts of forest loss and the prolonged consequences of its subsequent succession. This enhances our understanding of the evolving biophysical climate feedback from forest loss, providing valuable insights for climate mitigation and adaptation strategies as well as future climate predictions.[8,9,15] Moreover, forest loss can induce changes in the LST seasonal cycle, affecting both seasonal amplitude and phase. These changes have first-order ecological and societal effects, influencing biological cycles in plants, agricultural production, animal migration patterns, the spread of epidemics, and the estimation of long-term LST trends.[16–20]

However, existing studies on LST response to forest loss have primarily focused on static differences before and after forest cover change, known as the space-and-time scheme,[21–24] or on contrasts between equilibrium forest and non-forest cover, referred to as the space-for-time analogy.[5,7,25,26] The former method conflates the abrupt LST response to forest loss with the gradual response associated with subsequent land succession, rendering results highly dependent on the arbitrary selection of static post-loss timeframes. The latter method overlooks the temporal dynamics of the LST response following loss. The static post-loss states or equilibrium non-forest cover examined in these studies may represent different evolutionary stages, leading to contradictions in the signs and magnitudes of the LST response to forest loss,[7,11,21,26] as well as in the asymmetric impacts of forest gain and forest loss.[15,27] Recent studies focusing on specific types of forest loss (such as forest fires) or conducted at regional scales have identified dynamic LST responses following forest disturbances.[13,14] However, these analyses fail to account for discrepancies in LST responses induced by various types of forest loss,[22,27] and our understanding of how global forest loss and its subsequent succession affect local climate remains limited. Additionally, changes in the seasonal LST signal caused by forest loss in the existing literature are generally characterized by variations in monthly or seasonal data, which conflate shifts in the seasonal cycle with changes in the annual mean (Figure S1).[28] The extent and underlying causes of changes in seasonal cycle due to forest loss remain underexplored.

This study aims to reveal the dynamic LST responses to forest loss by disentangling the abrupt, gradual, and seasonal changes in LST, with a specific focus on whether abrupt changes are attenuated or enhanced post-loss. We propose an observation-driven method that integrates an improved change-detection algorithm with the space-and-time scheme. This method first decomposes the LST time series into a trendC and a seasonal component, then quantifies dynamic LST responses through abrupt and gradual changes in LST trendC as well as shifts in seasonal amplitude and phase. By utilizing satellite-observed LST time series, we assess LST responses following various types of forest loss, including deforestation driven by commodity production through

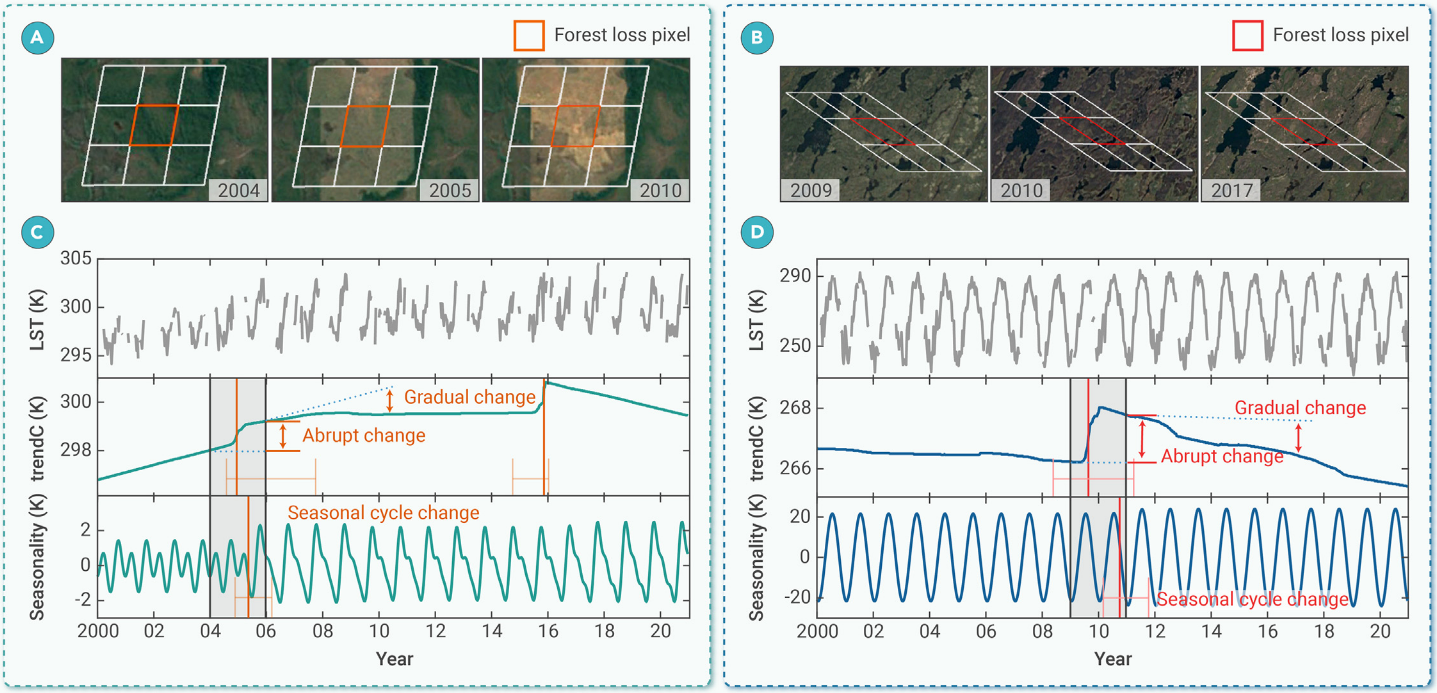

Figure 1. Illustration of the LST response to forest loss on multiple timescales High-spatial-resolution image sequences from Google Earth depict deforestation for crop expansion in the Brazilian Amazon in 2005 (A) and a fire in northern Saskatchewan Province, Canada in 2010 (B). White, red, and orange boxes represent MODIS pixels with a spatial resolution of 1 km. The sampled forest-loss pixels are centered at 49.66°W, 10.21°S and 102.95°W, 57.75°N, respectively. (C and D) Corresponding change-detection results for the LST time series. The change-detection method decomposes the LST time series into trend (trendC) and seasonal (seasonality) components and detects abrupt changes, marked by vertical red lines. Light red and orange vertical and horizontal lines represent the confidence intervals of the detected abrupt changes. The shaded areas between the black vertical lines indicate the time window during which forest loss occurred (see materials and methods).

permanent conversion from forest to cropland (cCRO) and by urbanization (URB), as well as forest disturbances such as temporary forest loss for agriculture with subsequent regrowth (shiftAG), forestry management, and fire. Additionally, we investigate the underlying biophysical mechanisms driving these responses.

MATERIALS AND METHODS

Our assessment of biophysical impacts of forest loss based on the proposed enhanced observational method using satellite data includes four steps: (1) change detection in the LST time series, (2) extraction of paired pixels, (3) quantification of LST response to forest loss, and (4) exploration of biophysical mechanisms driving LST changes based on the temperature-response model (Figure S2).

Data preparation

The Moderate Resolution Imaging Spectroradiometer (MODIS) time-series LST data from 2000 to 2020 were used to quantify the effect of forest loss on the local climate. First, we calculated daily mean LST using Xing's compositing algorithm, which integrates four daily LST observations from MOD11A1 and MYD11A1 products.[29] The estimated daily mean LST demonstrates high accuracy compared to *in situ* observations and strong effectiveness in deriving parameters in annual temperature cycle models, highlighting its value for time-series analysis. Each calendar month was divided into three groups of 10-day intervals. The LST time series, with a temporal resolution of 10 days and a spatial resolution of 1 km, was then generated by averaging daily LST within each group.

Surface shortwave albedo and evapotranspiration (ET) are two primary biophysical factors that regulate local climate by altering radiative forcing and land-atmosphere interaction efficiency. To ensure consistency with the MODIS LST data in spatial resolution, temporal resolution, and time span, and to minimize potential errors associated with multi-source data integration, this study utilized MODIS ET and albedo products (2000–2020) to investigate the mechanisms underlying the LST response. Albedo was calculated as the mean value of the black-sky albedo and white-sky albedo in the shortwave bands, as provided by the MCD43A3 product, which has a spatial resolution of 500 m and a daily temporal resolution. ET observations were obtained from the MOD16A2 product, which provides global terrestrial ET at an 8-day temporal resolution and a spatial resolution of 500 m. Both time-series albedo and ET data were resampled to a 1-km spatial resolution and a 10-day temporal resolution.

Data for forest cover change were obtained from high-resolution global forest maps of the twenty-first century, derived from time-series analysis of Landsat imagery. These maps provide global forest extent in 2000, as well as forest loss and gain from 2000 to 2021, at a spatial resolution of 30 m. The forest loss percentage was calculated by multiplying the forest cover percentage in 2000 by each loss pixel's area. Based on the global forest-loss map, Curtis et al. classified its drivers to distinguish permanent deforestation from temporary disturbances from 2001 to 2015, achieving an overall accuracy of 89%.[30] Their results demonstrate that global forest loss can be attributed to deforestation such as cCRO (accounting for 27%), as well as disturbances driven by forestry (26%), shiftAG (24%), and wildfires (23%). To align with the MODIS time-series data, we calculated the average forest loss percentage, forest gain percentage, and forest-cover percentage at a 1-km spatial resolution. Additionally, the forest-loss map and its associated drivers were aggregated to a 1-km resolution using the mode value (Figure S3).

The land-cover map and elevation data are used to select paired pixels, consisting of a forest-loss pixel and its adjacent forest-cover pixels. The ESA-CCI_LC data provide a consistent series of global land-cover maps at a 300-m spatial resolution on an annual basis from 1992 to 2020.[31] To identify valid background pixels with stable forest cover, we reclassified the original ESA-CCI_LC data into seven main land-cover types, including cropland, forest, natural vegetation, urban, bare land, water, and ice/snow.[32] Then the reclassified data were upscaled to a 1-km spatial resolution. Elevation data with a 1-km spatial resolution were aggregated from the Version 4 Shuttle Radar Topography Mission (SRTM) with an original 90-m resolution.

Additional auxiliary data include annual solar incident radiation data and broadband emissivity, which were used to calculate the equivalent changes in LST caused by altered albedo and evapotranspiration. Solar incident radiation was obtained from the monthly aggregate ERA5 atmospheric reanalysis product, which combines forecast models and data-assimilation systems to generate a long-term series of terrestrial and oceanic climate variables. Broadband surface emissivity was estimated using an empirical equation based on emissivity for MODIS bands 28, 30, and 32.[33]

To better identify consistent patterns and regional differences in LST responses to forest loss, we divided the study area based on latitude and Köppen climate types. Latitude was used to account for significant differences in LST seasonal cycles across latitudinal zones and hemispheres, as opposing seasonal signals between hemispheres could otherwise offset each other. Köppen climate types were used to capture variations in energy balance and land-atmosphere interactions, which differ across climate zones, forest types, and snow-cover conditions. In this study,

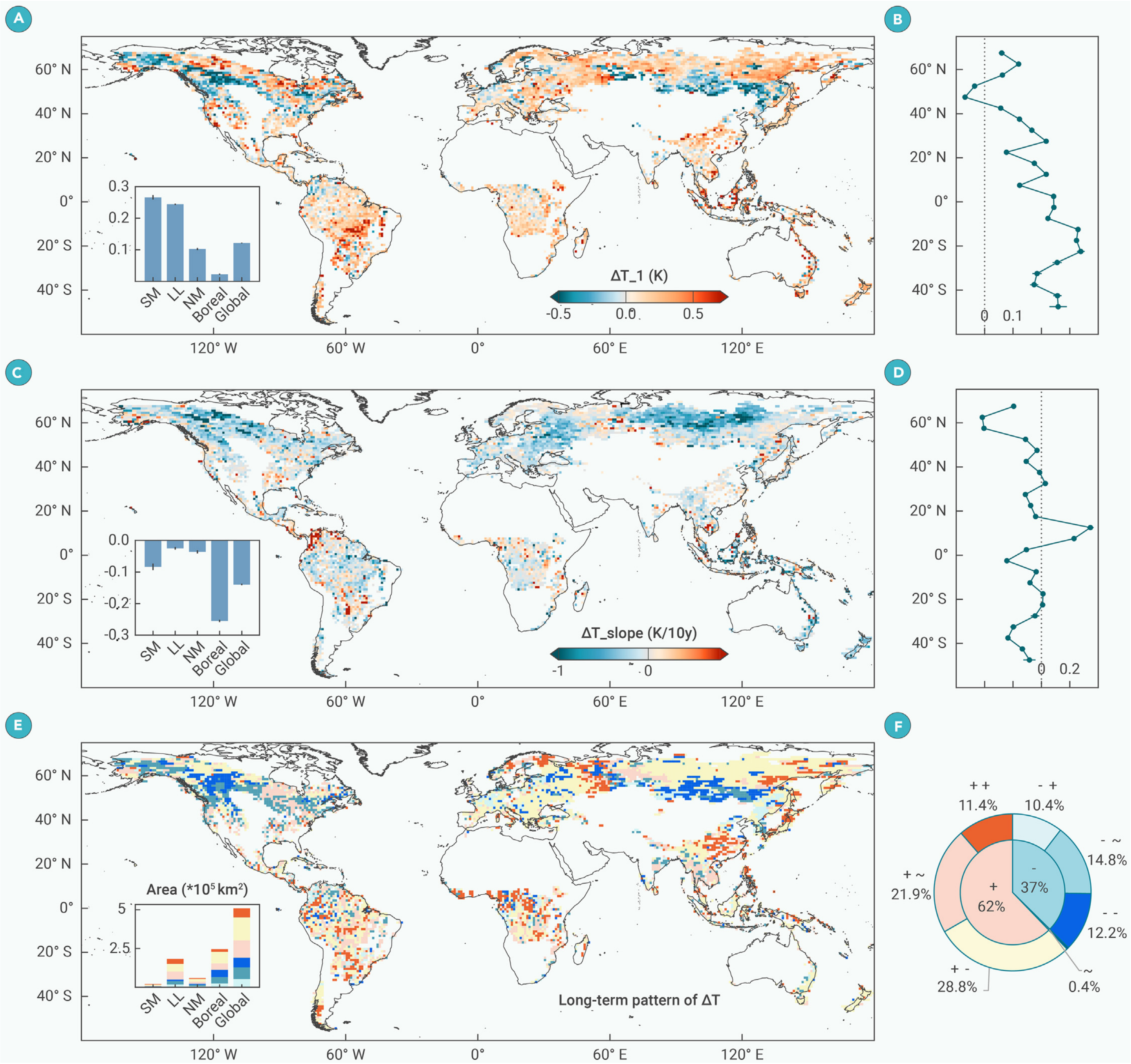

Figure 2. Changes in LST trendC (ΔT) caused by forest loss (A) Spatial distribution of abrupt changes in trendC (ΔT_1). (B) Zonal mean values of ΔT_1 averaged into 5° latitude bins. (C) Spatial distribution of linear slope of the gradual changes in trendC (ΔT_slope). (D) Zonal mean values of ΔT_slope averaged into 5° latitude bins. (E) Spatial distribution of the temporal pattern of ΔT. (F) Area proportions of different change patterns. Points in (E) are labeled as "enhanced warming" (+ +), "attenuated warming" (+ –), "persistent warming" (+ ∼), "enhanced cooling" (– –), "attenuated cooling" (– +), and "persistent cooling" (– ∼) based on the interaction between ΔT_1 and ΔT_slope. The first sign represents ΔT_1: "∼" indicates insignificant changes when $|\Delta T\_1|$ is smaller than the margin of error (0.001 K) for a 95% confidence interval of the globally averaged ΔT_1, "+" indicates warming with $\Delta T\_1 > 0.001$ K, and "–" indicates cooling with $\Delta T\_1 < -0.001$ K. The second sign represents ΔT_slope; similarly, "∼" indicates insignificance with $|\Delta T\_slope| < 0.002$ K/10y, "+" indicates warming with $\Delta T\_slope > 0.002$ K/10y, and "–" indicates cooling with $\Delta T\_slope < -0.002$ K/10y. All grids are spaced at 1° in both latitude and longitude. The embedded bar figures show the zonal averaged values or zonal areas across the southern mid-latitudes (SM), low latitudes (LL), northern mid-latitudes (NM), boreal regions, and global regions. Error bars in bar and line figures represent 95% confidence intervals of zonal averaged values.

the forest-loss areas were divided into four regions: the southern mid-latitudes (SM), covering the area between 60° S and 20° S; the low latitudes (LL), spanning the area between 20° S and 20° N; the boreal region, defined as the boreal climate zone above 20° N according to the Köppen-Geiger world map; and the northern mid-latitudes (NM), which comprise non-boreal areas above 20° N (Figure S4). Additionally, all data were reprojected to sinusoidal projection coordinates.

Change detection in LST time series

The improved breaks for additive seasonal and trend (BEAST) method employs a multi-linear function and multiple second-order harmonic functions to simulate the trendC and seasonality in the LST time series as well as to identify the abrupt changes in these components. This method has been proven effective in detecting land cover changes by using time-series LST.[12,34] The improved BEAST is expressed as follows:

$$LST_t = T_t + S_t + e_t, t = 1, \ldots, n,$$

$$T_t^i = a_i + b_i \cdot t, \left(\tau_{i-1}^* < t \le \tau_i^*\right), i = 1, \ldots, m + 1$$

$$S_t^j = \sum_{h=1}^{2}\left[\gamma_{j,h} \cdot \sin\left(\frac{2\pi ht}{\lambda}\right) + \delta_{j,h} \cdot \cos\left(\frac{2\pi ht}{\lambda}\right)\right], \left(\tau_{j-1}^{\#} < t \le \tau_j^{\#}\right), j = 1, \ldots, q + 1$$

(Equation 1)

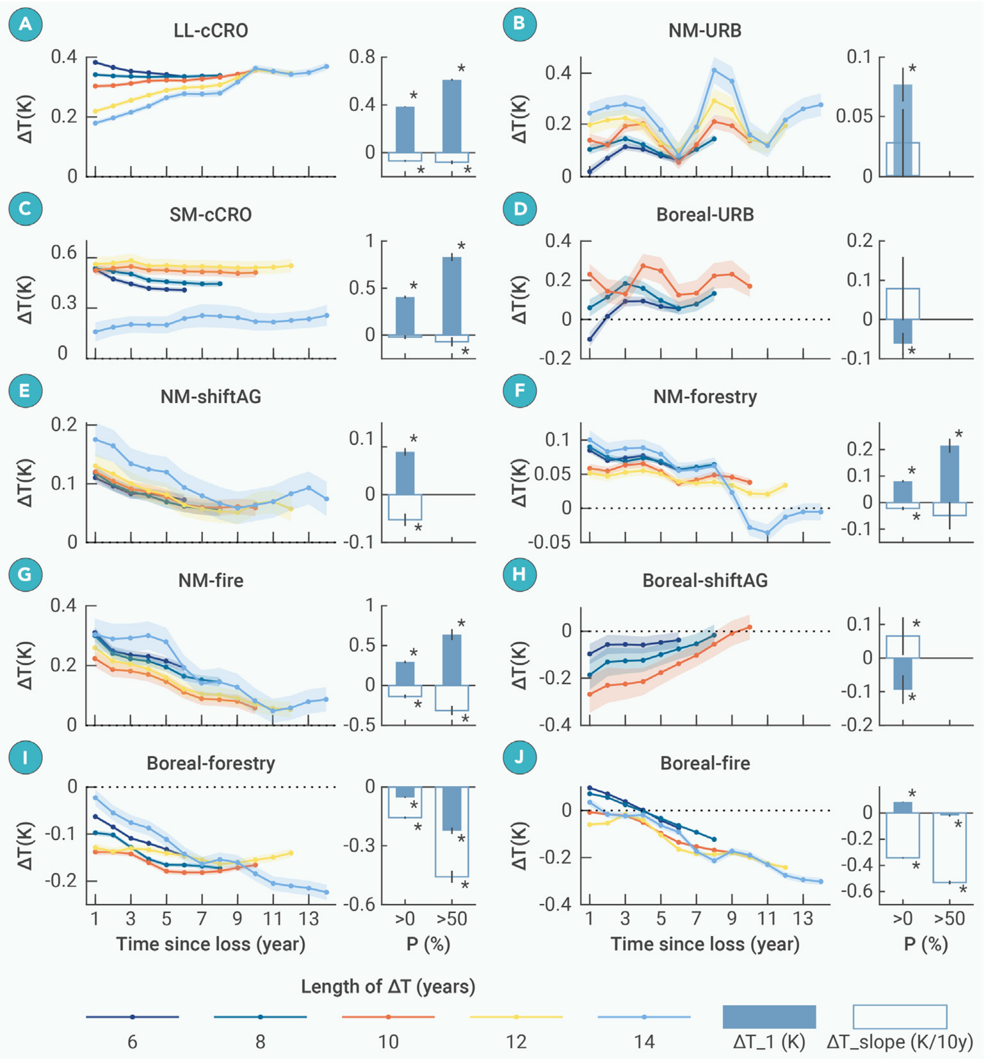

Figure 3. Temporal dynamics of ΔT from 1 to 14 years after the forest loss across five loss types Commodity-driven conversion from forest to cropland (cCRO; A and C), urbanization (URB; B and D), shifting agriculture (shiftAG; E and H), forestry (F and I), and fire (G and J). Forest loss occurred between 2005 and 2015. The titles of subfigures (A–J) indicate the climate zone (e.g., SM, LL, NM, and boreal zone) and forest-loss type. On the left side, colored lines represent ΔT dynamics over varying durations (6–14 years) with loss percentages (P) of greater than 0%. Shaded areas around the lines represent 95% confidence intervals. On the right side, bar charts display zonal mean values of abrupt changes in trendC (ΔT_1) and the linear slope of the gradual changes in trendC (ΔT_slope) for P > 0% and P > 50%, respectively. Error bars represent 95% confidence intervals. Asterisk (*) indicates zonal mean values that are statistically significant at the 5% level based on a t test against zero.

where the time-series LST (LST_t) is treated as the sum of trendC (T_t), seasonality (S_t), and the remainder (e_t). t is the time phase and n is the number of LST observations. The trendC is simulated by $m + 1$ linear segments (T_t^i) divided by m trend breakpoints ($\tau_1^*, \ldots, \tau_m^*$). a_i and b_i are the linear function-related parameters at segment i. Similarly, LST seasonality is modeled using multiple second-order harmonic functions (S_t^j). $q + 1$ is the number of seasonal segments and $\tau_1^\#, \ldots, \tau_q^\#$ are seasonal breakpoints. $\gamma_{j,h}$ and $\delta_{j,h}$ are the parameters related to the harmonic function at segment j. λ is the period of the LST time series (36 in this study).

As a first step, we applied the improved BEAST to the MODIS LST time series to decompose LST into trendC and seasonality while detecting the three most significant abrupt changes (i.e., m = q = 3). Change detection results from the three sample sites confirm that the improved BEAST, which utilizes 10-day time-series data, effectively captures both abrupt and gradual changes in LST trendC and seasonal variations following forest loss, even under conditions of poor data quality, such as data loss and significant fluctuations (Figures S5–S7). These findings also highlight a distinct advantage over annual time-series data. Figure S8 shows the global distribution of the number of abrupt changes detected in LST trendC and seasonality.

Paired pixels extraction

Forest-loss pixels selection. The MODIS LST time series used in our study is highly consistent after 2002. To ensure the availability of at least two years of pre-loss data, our assessment focuses on forest loss occurring between 2005 and 2015. We first identified forest-loss pixels associated with abrupt changes in LST. The criteria for matching detected abrupt changes in LST with forest-loss events are as follows:

(1) Forest loss occurs within the confidence interval of the detected abrupt changes in LST.
(2) The time difference between the forest loss and abrupt LST changes does not exceed two years.

For a given pixel, if the detected abrupt changes match a forest-loss event, the forest-loss window is defined as the union of the year in which abrupt changes occurred in LST trendC or seasonality and the year of the forest-loss event. Consequently, the study period for this pixel spans from one year before the window to the next detected abrupt change or the end of the time series, enabling the exploration of LST response evolution for up to 14 years. As illustrated in Figure S9, a sample pixel experienced deforestation in 2005, accompanied by abrupt changes in the LST trendC in 2005 and in seasonality in 2006. Thus, the forest-loss window for this pixel spans from early 2005 to late 2006. To investigate temporal changes in LST, we focus on the period from one year before the loss to the next abrupt change (i.e., the start of the confidence interval of the next abrupt change), which may be related to other disturbances. For the sample pixel in Figure S9, LST variations from 2003 to 2014 were analyzed.

Background pixel selection. Under the assumption that climate-variability remain consistent at a regional scale, we estimate the climate signal of a forest-loss pixel by analyzing LST changes in adjacent areas (i.e., background pixels) with stable forest cover. Valid background pixels serving as controls for each forest-loss pixel should (1) be located within a search window of 25–50 km from the forest-loss pixel; (2) maintain stable forest land cover, with a forest-change percentage lower than the minimum of 2% and the loss percentage of the forest-loss pixel; and (3) have an elevation difference of less than 100 m compared to the forest-loss pixel. While absolute LST values may vary significantly with elevation, temporal LST changes remain synchronized because both forest-loss pixels and their background pixels are exposed to the same climatic conditions. This is supported by a comparative analysis of the direct LST values at different elevations and elevation-corrected LST values.[15] Moreover, the number of valid background pixels must exceed 5% of all background pixels to ensure robust statistical analysis. Otherwise, the paired samples were excluded from our assessment.

Quantification of LST response to forest loss

The dynamic responses of LST to forest loss are quantified by changes in two LST components: trendC and seasonality, including abrupt changes in LST trendC, gradual changes in the post-loss LST trendC trajectory, and changes in seasonal amplitude and phase. For each forest-loss pixel, LST changes are calculated as the difference between the observed LST variations of the forest-loss pixel and those of its valid background pixels. Figure S9 illustrates this calculation.

The time-series changes in LST trendC (ΔT) following forest loss are expressed by Equation 2, which captures the temporal dynamics of the LST response.

$$\Delta T = \delta T^{o} - \delta T^{bg}, \quad \delta T^{o} = T^{o,after} - T\_1^{o,before} \qquad \text{(Equation 2)}$$

where o and bg represent the target forest-loss pixel and its background pixels, respectively. δT^o is the difference in annual trendC of the forest-loss pixel between post-loss ($T^{o,after}$) and one year before loss ($T\_1^{o,before}$). Similarly, δT^{bg} is the mean difference in

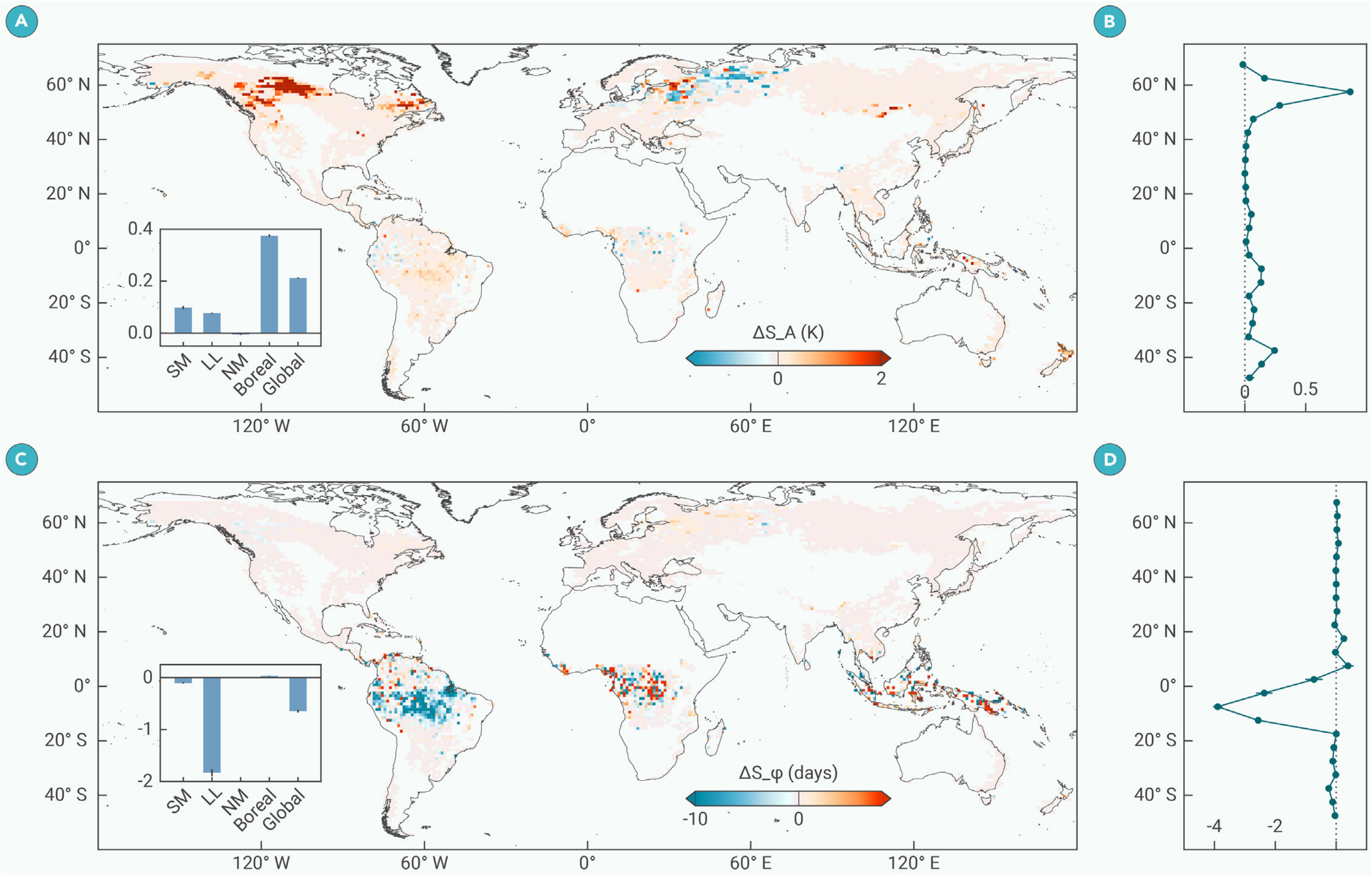

Figure 4. Changes in seasonal amplitude (ΔS_A) and seasonal phase (ΔS_ϕ) caused by forest loss (A and C) Spatial distributions of ΔS_A and ΔS_ϕ, respectively. Line charts (B and D) on the right side represent zonal mean values averaged into 5° latitude bins. The embedded bar charts show the zonal mean values across the SM, LL, NM, boreal regions, and global regions. Error bars in bar and line figures represent 95% confidence intervals of zonal averaged values.

trendC among the background pixels.[15,35] Since the distance between the forest-loss pixels and their background pixels may influence the quantification of background LST changes (δT^{bg}), we tested the inverse-distance-weighting (IDW) method to improve the robustness of our assessment:

$$\delta T^{bg} = \frac{\sum_{bgi=1}^{bgn} \frac{\delta T^{bgi}}{d_{bgi}}}{\sum_{bgi=1}^{bgn} \frac{1}{d_{bgi}}} \quad \text{(Equation 3)}$$

where bgn is the number of valid background pixels, and d_{bgi} is the distance between the forest-loss pixel and background pixel bgi. The results indicate that the method of background signal extraction does not affect the conclusion (Figure S10).

To further quantify the ΔT dynamics, we calculate both abrupt and gradual changes in trendC. The abrupt changes, denoted as ΔT_1, represent ΔT one year after the loss. The gradual changes are quantified by the change in the linear slope of the post-loss LST trendC (ΔT_slope):

$$\Delta T_{slope} = \delta T_{slope}{}^{o} - \delta T_{slope}{}^{bg},\ \delta T\_slope^{o} = T\_slope^{o,after} - T\_slope^{o,before} \quad \text{(Equation 4)}$$

where δT_slope^{o} is the difference in linear slope of the trendC for forest-loss pixel between post ($T\_slope^{o,after}$) and pre-loss ($T\_slope^{o,before}$). Similarly, δT_slope^{bg} is the difference between $T\_slope^{bg,after}$ and $T\_slope^{bg,before}$ for background pixels. $T\_slope^{o,before}$ is assumed to be equal to $T\_slope^{bg,before}$ due to identical climate signals.

The changes in seasonal component (ΔS) are calculated by

$$\Delta S = \delta S^{o} - \delta S^{bg}, \quad \delta S^{o} = S^{o,after} - S^{o,before} \quad \text{(Equation 5)}$$

where δS^{o} is the change in LST seasonal component across different months for the forest-loss pixel between post ($S^{o,after}$) and pre-loss ($S^{o,before}$). δS^{bg} is the change in the seasonal component for the background pixel. To further quantify seasonal changes, we calculate the change in amplitude (ΔS_A) and phase (ΔS_ϕ) of the LST seasonal cycle:

$$\begin{aligned} \Delta S\_A &= \delta S\_A^{o} - \delta S\_A^{bg},\ \delta S\_A^{o} = S\_A^{o,after} - S\_A^{o,before} \\ \Delta S\_\phi &= \delta S\_\phi^{o} - \delta S\_\phi^{bg},\ \delta S\_\phi^{o} = S\_\phi^{o,after} - S\_\phi^{o,before} \end{aligned} \quad \text{(Equation 6)}$$

where δS_A^{o} is the change in seasonal amplitude for the forest-loss pixel, calculated as the difference in seasonal amplitude between post ($S\_A^{o,after}$) and pre-loss ($S\_A^{o,before}$). Here, seasonal amplitude is defined as the difference between maximum and minimum LST seasonality. The change in the seasonal amplitude for the background pixel (δS_A^{bg}) is calculated similarly. δS_ϕ^{o} and δS_ϕ^{bg} are the changes in the seasonal phase. S_ϕ is estimated by fitting a first-order harmonic function to LST seasonal component, which determines the time of year when the fitted curves reach their maximum. Additionally, to ensure comparability of ΔS and ΔS_A across different latitudinal zones, we calculate the relative changes by dividing ΔS by $S^{o,before}$ and dividing ΔS_A by $S\_A^{o,before}$, respectively.

Temperature-response model

The mechanism underlying the biophysical climate impact of forest loss can be expressed as LST response to changes in biophysical factors associated with forest loss based on the surface-energy-balance equation:[7]

$$\Delta LST = \frac{1}{4\varepsilon\sigma LST^{3}}[-SW\downarrow\cdot\Delta\alpha + (1-\alpha)\cdot\Delta SW\downarrow + \Delta LW\downarrow - \Delta LE - \Delta H - \Delta G] \quad \text{(Equation 7)}$$

where $\frac{1}{4\varepsilon\sigma LST^{3}}$ is the first-order Taylor expansion term of upward long-wave radiation, ε is the broadband surface emissivity, σ is the Stefan-Boltzmann constant, and LST is the pre-loss mean LST for the forest-loss pixel calculated as the mean value in 2003. $SW\downarrow$ is the downwelling shortwave solar radiation, α is the surface albedo, $LW\downarrow$ is the downwelling long-wave

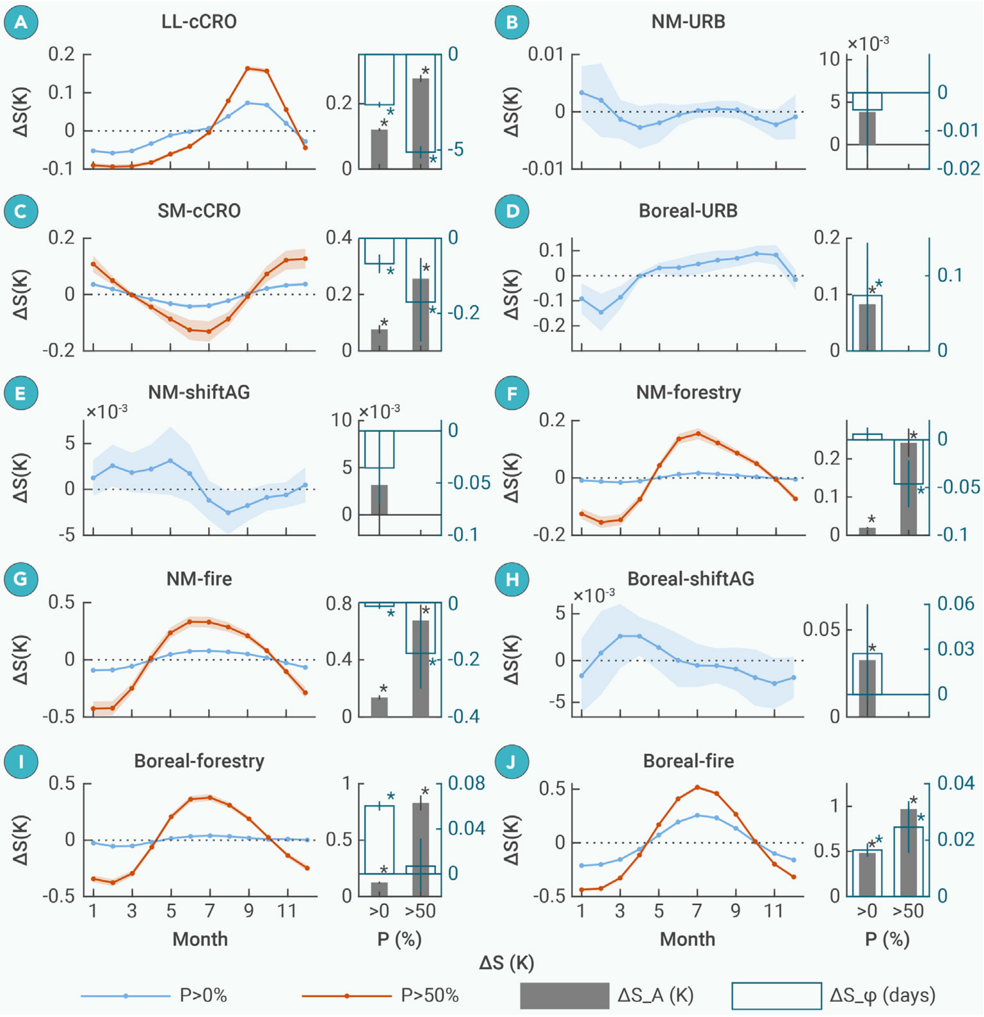

Figure 5. Changes in seasonal components in different months (ΔS), seasonal amplitude (ΔS_A), and seasonal phase (ΔS_ϕ) caused by forest loss driven by cCRO, URB, shiftAG, forestry, and fire across different climate zones The titles of subfigures (A–J) indicate the climate zone and forest-loss type. On the left side, colored lines represent **ΔS** for loss percentage (P) greater than 0% (blue) and 50% (red), respectively. Shaded areas around the lines represent 95% confidence intervals. Bar charts on the right side represent the zonal mean values of **ΔS_A** (black) and **ΔS_ϕ** (blue) for P > 0% and P > 50%, respectively. Error bars represent 95% confidence intervals. Asterisk (*) indicates zonal mean values that are statistically significant at the 5% level based on a t test against zero.

radiation, *LE* is latent heat flux, *H* is sensible heat flux, and *G* is soil heat flux. The terms $\Delta SW\downarrow$ and $\Delta LW\downarrow$ are assumed negligible, as both the forest-loss pixel and its background pixels receive the same amount of $SW\downarrow$ and $LW\downarrow$. Additionally, minor terms such as changes in G and ε are also considered negligible.[7,36–39] Temperature-response models that explicitly account for aerodynamic resistance and energy partitioning were not employed due to the limited spatial resolution of key meteorological data, such as air temperature, atmospheric specific humidity, and wind speed.[7,40,41] Therefore, the LST response to forest loss can be expressed as the sum of a radiative forcing term resulting from $\Delta\alpha$ and an energy-redistribution term contributed by ΔLE and ΔH:

$$\Delta LST = \Delta LST_{\alpha} + \Delta LST_{LE} + \Delta LST_{H},$$
$$\Delta LST_{\alpha} = \frac{1}{4\varepsilon\sigma LST^{3}}\cdot(-SW\downarrow\cdot\Delta\alpha) \qquad \text{(Equation 8)}$$
$$\Delta LST_{LE} = \frac{1}{4\varepsilon\sigma LST^{3}}\cdot(-\Delta LE)$$
$$\Delta LST_{H} = \frac{1}{4\varepsilon\sigma LST^{3}}\cdot(-\Delta H)$$

where ΔLST_{α}, ΔLST_{LE}, and ΔLST_{H} are the equivalent LST responses to $\Delta\alpha$, ΔLE, and ΔH, respectively. ΔLE is calculated by converting the ΔET from mm/10 days to W/m^2. $SW\downarrow$ is the pre-loss mean solar incident radiation in 2003 for the target forest-loss pixel.

Our method decomposes ΔLST into changes in trendC (ΔT) and seasonality (ΔS); therefore, the mechanisms driving ΔT and ΔS can be quantified by replacing *LST* in Equation 8 with trendC or seasonality, respectively. The equation is as follows:

$$\Delta T = \Delta T_{\alpha} + \Delta T_{LE} + \Delta T_{H}$$
$$\Delta S = \Delta S_{\alpha} + \Delta S_{LE} + \Delta S_{H} \qquad \text{(Equation 9)}$$

where ΔT_{α} and ΔT_{LE} are the equivalent temporal changes in LST trendC, induced by altered trend components in α and *LE*, respectively. Similarly, ΔS_{α} and ΔS_{LE} are the equivalent LST seasonal changes driven by altered seasonal components in α and *LE*. Using the improved BEAST, we first decomposed the time-series α and *ET* into trendC and seasonality. Change-detection results from three selected sample sites confirmed the reliability and effectiveness of BEAST in tracking albedo dynamics (Figure S11). Then changes in trend and seasonal α and *LE* were calculated as the differences between forest-loss pixels and their background pixels over the defined study period. These changes were then converted into equivalent changes in both ΔT and ΔS.

RESULTS

Abrupt and gradual changes in LST due to forest loss

Figure 2 shows the abrupt (ΔT_1; Figures 2A and 2B) and gradual (ΔT_slope; Figures 2C and 2D) changes in LST trendC (ΔT), as well as the temporal pattern of ΔT (Figures 2E and 2F) caused by forest loss. ΔT_1 represents the trendC difference between one year before and after the loss after eliminating background climate variation. It captures the immediate climatic response. ΔT_slope is the difference in linear slope of post-loss trendC trajectories between forest-loss pixels and their background control pixels, reflecting the gradual impact of post-loss land-cover change. The temporal pattern of ΔT is determined by the interaction between ΔT_1 and ΔT_slope.

Forest loss produces a marked abrupt warming, with a global ΔT_1 of +0.12 K (Figure 2A). The ΔT_1 exhibits distinct latitudinal patterns (Figure 2B). Between 50° S and 45° N, ΔT_1 is positive, with the strongest warming observed in the cCRO regions of the Amazon around 20° S. From 45° N to 55° N, immediate cooling, indicated by a negative ΔT_1, is associated with extensive forestry operations. North of 55° N, immediate warming (positive ΔT_1) is evident due to extensive forest fires (Figure S3). Over the long term, the global average ΔT exhibits a decreasing trend, with a ΔT_slope of −0.14 K per decade (10y) following the loss (Figure 2C). The most rapid decrease (−0.26 K/10y) occurs in boreal regions, while the smallest decrease (−0.03 K/10y) is observed in the LLs (Figure S4). Between 5° N and 15° N, where forest loss is primarily driven by cCRO and shiftAG (Figure S3), ΔT shows an increasing trend (Figure 2D). The temporal patterns of ΔT can be classified into seven main categories: enhanced warming (++), abrupt warming (+ ∼), attenuated warming (+ −), attenuated cooling (− +), abrupt cooling (− ∼), enhanced cooling (− −), and insignificant changes (∼) (Figures 2E and 2F). Globally, the dominant pattern is attenuated warming (28.8%), indicating initial warming followed by a decreasing trend (Figure 2F). These patterns differ across climate regions (Figure 2E; Table S1). In the boreal region, the predominant patterns include attenuated warming and enhanced cooling. In the SM latitudes, attenuated warming is prominent. In the LLs, ΔT exhibits diverse temporal patterns, including abrupt warming, attenuated warming, and enhanced warming.

We further compare the ΔT dynamics resulting from different types of forest loss. Figure 3 presents multiple post-loss trajectories of ΔT, selected based on adequate sample size and varying time-series lengths (Figure S12), which

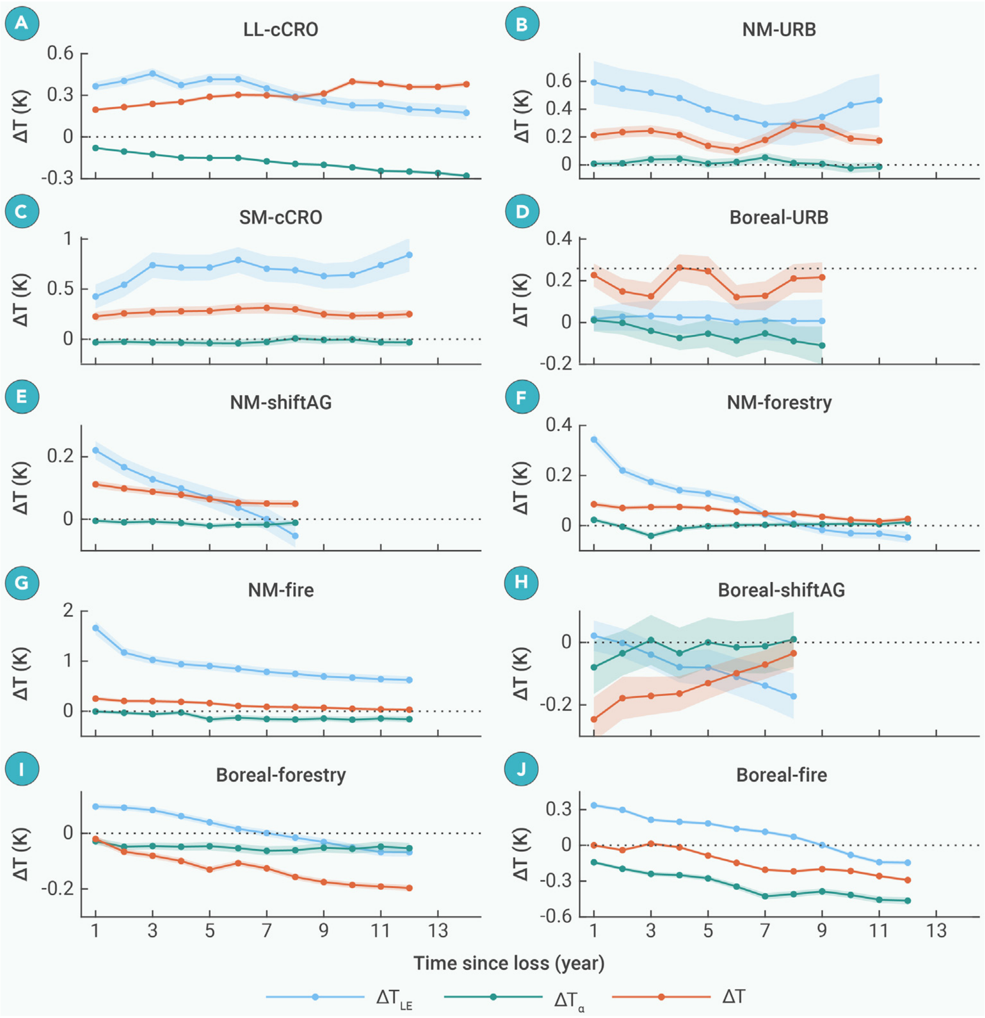

Figure 6. Comparison of the equivalent temporal changes in LST (ΔT) induced by changes in LE (ΔT_{LE}) and albedo (ΔT_{α}) to the observed ΔT The titles of subfigures (A–J) indicate the climate zone and forest-loss type. The shaded areas represent 95% confidence intervals.

enhances the reliability of ΔT evolutions. Each trajectory ensures sample consistency throughout its time series. Permanent forest loss driven by cCRO emerges as the primary contributor to warming, with ΔT_1 values of +0.39 K and +0.41 K in the LL and SM latitudes, respectively (Figures 3A and 3C). Notably, deforestation for cCRO in the SM latitudes exhibits the highest warming sensitivity, with a maximum ΔT_1 (+0.83 K) when loss percentage (P) exceed 50% (Figure 3C). Over the long term, ΔT shows an increasing trend in sufficiently extended sequences, such as those longer than 10 years, while shorter sequences show a stable or even decreasing trend (Figures 3A and 3C). This difference may arise from variations in sample composition across time series of different lengths and inconsistencies in agricultural management. URB generally leads to enhanced warming with the highest positive ΔT_slope among all forest-loss types (Figures 3B and 3D). URB in the NM latitudes results in a ΔT_1 of +0.08 K and a ΔT_slope of +0.03 K/10y (Figure 3B). In the boreal region, URB may initially trigger cooling ($\Delta T\_1 < 0$), but this effect is temporary, with a ΔT_slope of +0.08 K/10y (Figure 3D).

Forest disturbances that occurred in the NM latitudes cause attenuated warming. Specifically, shiftAG, forestry, and fire result in an abrupt positive ΔT followed by a recovery trend ($\Delta T\_1 > 0$ and $\Delta T\_slope < 0$), indicating that warming diminishes as forests recover (Figures 3E–3G). Among these disturbances, fire exhibits the most pronounced positive ΔT_1 (+0.30 K) and the steepest decreasing trend (ΔT_slope = −0.14 K/10y), as well as the highest sensitivity (ΔT_1 = +0.64 K and ΔT_slope = −0.31 K/10y when P > 50%). A similar pattern of attenuated warming is observed following disturbances in the low and mid-latitudes, although shiftAG shows persistent warming. This persistence is likely due to the misclassification of much cCRO as shiftAG in these regions (Figure S13).[30] In the boreal zone, shiftAG results in a recovery dynamic of ΔT (i.e., attenuated cooling) due to documented forest return (Figure 3H). In contrast, forestry and fire do not exhibit a recovery dynamic. Forestry leads to enhanced cooling, with a ΔT_slope of −0.16 K/10y (Figure 3I). Compared to forestry, fire initially causes attenuated warming but later transitions to enhanced cooling, exhibiting a more pronounced decreasing trend (ΔT_slope = −0.34 k/10y) and higher sensitivity (ΔT_slope = −0.53 K/10y when P > 50%) (Figure 3J). The continued deviation of ΔT from zero due to forestry and fire in the boreal region suggests lower forest resilience compared to that in the low and mid-latitudes.

Changes in the LST seasonal cycle due to forest loss

The changes in LST seasonal amplitude (ΔS_A) and seasonal phase (ΔS_ϕ) caused by forest loss are shown in Figure 4. On average, forest loss amplifies the seasonal amplitude by +0.21 K, with the most pronounced effects observed in the boreal region (ΔS_A = +0.37 K) (Figures 4A and 4B). Concurrently, forest loss advances the seasonal phase by 0.6 days, with the most significant shifts occurring in LLs (ΔS_ϕ = −1.8 days) (Figures 4C and 4D).

Due to the substantial divergence in the magnitude of seasonal changes among different loss types and climate zones, Figure 5 illustrates the changes in LST seasonal component across different months (ΔS), as well as ΔS_A and ΔS_ϕ, for the selected forest-loss regions shown in Figure 3. Deforestation for cCRO in the LLs enhances seasonal amplitude (ΔS_A = +0.12 K) by increasing the seasonal values from July to November and decreasing them from December to June, with the largest relative change (+6.4%) and sensitivity (+14.3% when P > 50%) (Figures 5A and S14). URB also amplifies seasonal amplitude in the boreal region but with a smaller magnitude (Figure 5D). Forest disturbances driven by forestry and fire lead to higher summer values and lower winter values, which is particularly evident in the boreal fire regions (Figures 5F, 5G, 5I, 5J, and S14). These regions exhibit the greatest ΔS_A (+0.5 K) and the highest sensitivity (+1.0 K when P > 50%), although the relative ΔS_A (+1.2%) is smaller than that of cCRO. In contrast, the ΔS_A values induced by URB in the NM latitudes and shiftAG are insignificant (Figures 5B–5E and 5H). Notably, ΔS exhibits a symmetric magnitude but opposite signs in summer and winter. This is because ΔS represents only the seasonal signals after eliminating trendC from the original time series, and its expected value is zero (see materials and methods).

The cCRO in the LLs results in the greatest early shifts in seasonal phase (ΔS_ϕ = −2.6 days) and sensitivity (−5.1 days when P > 50%) (Figure 5A). As latitude increases, the phase response transitions from advancement to delay, with progressively weaker effects. Specifically, cCRO in the SM latitudes, as well as shiftAG, forestry, and fire occurring in the NM latitudes, all induce early shifts in seasonal phase ($\Delta S\_\phi < 0$), although with smaller magnitudes compared to those in the LLs. Among these, only the shifts associated with cCRO and fire are statistically significant (Figures 5C, 5F, and 5G). In contrast, in the boreal region, URB, shiftAG, forestry, and fire result in slight delays in seasonal phase ($\Delta S\_\phi > 0$), with significant changes of +0.07, +0.03, +0.06, and +0.02 days, respectively (Figures 5D and 5H–5J).

Biophysical mechanism of the changes in LST

To elucidate the biophysical mechanisms underlying the LST response, Figure 6 compares the equivalent ΔT dynamics resulting from changes in latent

www.the-innovation.org

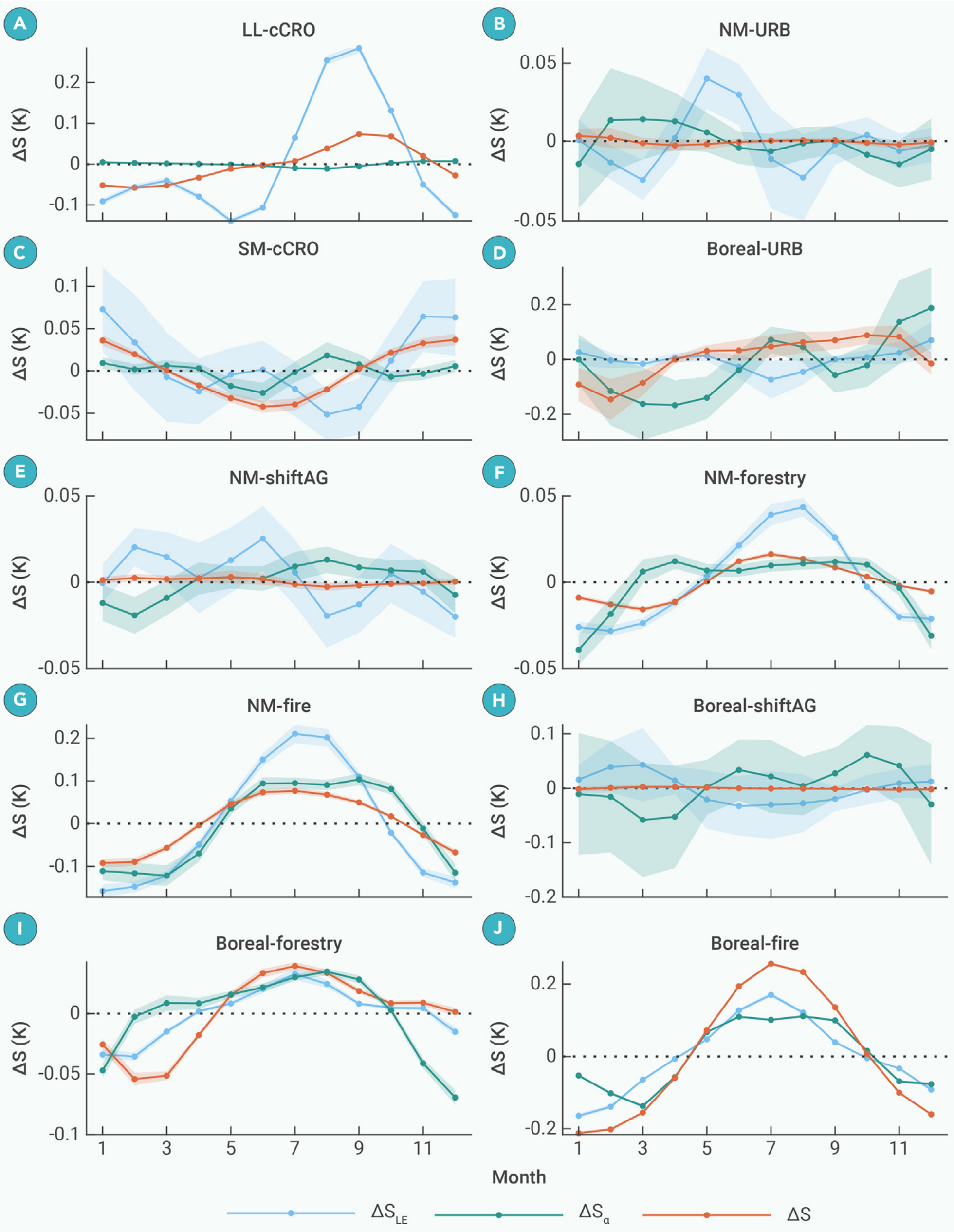

Figure 7. Comparison of the equivalent changes in LST seasonal components (ΔS) induced by changes in LE (ΔS_{LE}) and albedo (ΔS_{α}) with the observed ΔS in different months following the forest loss The titles of subfigures (A–J) indicate the climate zone and forest-loss type. The shaded areas represent 95% confidence intervals.

heat (ΔT_{LE}) and albedo (ΔT_{α}) to the observed ΔT. These ΔT trajectories were selected based on their adequate sample size and the longest available post-loss period (Figure S12).

For deforestation, early-stage warming induced by cCRO is primarily driven by a positive ΔT_{LE}, which is associated with reduced LE (Figures 6A and 6C). In later stages, the ΔT trajectory diverges from both ΔT_{LE} and ΔT_{α}, suggesting the influence of additional drivers, such as sensible heat.[42–44] URB-induced persistent warming in the NM latitudes is attributed to positive ΔT_{LE}. However, in the boreal region, this warming cannot be explained by ΔT_{LE} and may instead be related to overwhelming anthropogenic heat (Figures 6B and 6D).[45] For forest disturbances, the attenuated warming observed in mid-latitudes is controlled by the initial positive ΔT_{LE} and its subsequent recovery (Figures 6E–6G). Notably, the positive ΔT_{LE} following fire is significantly larger than that of forestry and shiftAG, and it does not return to zero within the study period. This indicates more pronounced warming and greater challenges in water usage restoration after the fire. Moreover, the magnitude and trend of ΔT_{LE} resulting from fire substantially exceed those of the observed ΔT, indicating that warming from LE reduction is partially counterbalanced by an increase in sensible heat due to reduced surface roughness.[46] In the boreal region, the contribution of ΔT_{α} becomes more pronounced compared to that in the low and mid-latitudes. The attenuated cooling from shiftAG is partially associated with the recovery of a negative ΔT_{α} (Figure 6H). The decreasing trend in ΔT following forestry is mainly attributed to the ΔT_{LE}, while ΔT_{α} plays a considerable role in early-stage net cooling (Figure 6I). For fire, ΔT_{LE} and ΔT_{α} contribute comparably to the decline in ΔT. The initial net neutral or even warming is driven by decreased LE, particularly during the summer season,[11,14] while subsequent net cooling is largely attributed to decreasing ΔT_{α} (Figure 6J).

The observed recovery dynamics of ΔT_{LE} following disturbances commence with vegetation regrowth, resulting in a higher LE than the initial post-loss state. After a period of recovery, the regenerated young trees, typically shorter in height and exhibiting higher leaf water potential, further enhance LE compared to the original tree cover.[27,47,48] In contrast, the albedo alteration resulting from boreal forestry and fire does not recover with vegetation regrowth. This is due to the limited vegetation cover in the early post-loss stage, which still exposes substantial bare surfaces that increase albedo, particularly under snow conditions. Moreover, albedo may not return to its pre-loss levels because of inherent differences between regenerated secondary forest and the original forest.[49–51] The progressive decrease in ΔT_{α} following fire can be attributed to two main factors. First, the persistent loss of canopy overstory due to the falling of dead snags in moderately and severely burned areas creates a more reflective surface for lying snow.[52] Second, the establishment of grass and shrub cover, combined with the partial loss of black carbon that initially coated soil surfaces and dead trees, leads to a progressive increase in albedo starting two years after the fire, especially in the summer under snow-free conditions.[53]

Figure 7 compares the equivalent changes in LST seasonality (ΔS) caused by LE (ΔS_{LE}) and albedo (ΔS_{α}) with observed ΔS. The mechanisms of ΔS exhibit distinct latitudinal patterns: ΔS_{LE} dominates the amplified seasonality in low and mid-latitudes, while the contribution of ΔS_{α} increases at higher latitudes. Specifically, cCRO in the LLs produces greater variability in LE seasonality, with increasing ΔS_{LE} from July to October and decreasing from November to June, while the contribution of the ΔS_{α} remains negligible (Figure 7A). In the SM latitudes, both LE and albedo contribute to the ΔS caused by cCRO (Figure 7C). In the boreal region, ΔS associated with URB is partially driven by albedo (Figure 7D). Disturbances driven by forestry and fire in the NM latitudes amplify ΔS, characterized by increased summer values and decreased winter values, primarily attributed to ΔS_{LE} (Figures 7F and 7G). In the boreal region, albedo plays a role comparable to LE (Figures 7I and 7J). Notably, forestry-induced ΔS_{α} peaks earlier than fire, indicating a more pronounced winter cooling following fire. In contrast, shiftAG exhibits negligible seasonal variation due to the offsetting effects of LE and albedo (Figures 7E and 7H). Regarding the LST seasonal phase shifts following forest loss, the earlier shifts in low and mid-latitudes are related to reduced heat capacity, which is modulated by soil moisture and vegetation density.[28,54] This is shown by substantial changes in seasonal LE (Figure 7). In the boreal zone, the delayed phase is attributed to reduced solar radiation forcing, particularly during winter.[28,55]

DISCUSSION

Our assessment recognizes that climate feedback from forest loss is climate specific, loss-type dependent, and time varying. By decoupling the abrupt impact of forest loss from the gradual impact of its subsequent succession, we reconcile inconsistencies in previous observational studies.[15,56] Previous analyses employing space-and-time schemes with long intervals between paired observations[21] or space-for-time analogies[7] likely underestimated the warming effect of forest disturbances in mid-latitudes due to their attenuated warming dynamics. Additionally, these estimates may have overlooked the warming or neutral climate feedback associated with fires in the boreal zone during the early stages of post-loss evolution.[11,14]

The pronounced trend of the LST response following temporary forest disturbances highlights the necessity of carefully considering the vegetation recovery trajectories when evaluating the climate effects of forest loss.[14,15] The enhanced cooling from forestry and fire in the boreal zone suggests their long-term and progressive influence on Earth's surface radiative budget, which is beneficial in mitigating local warming. The contrasting dynamics of LST responses to disturbances can be attributed to factors such as disturbance severity (e.g., loss percentage), snow conditions, post-loss regrowth vegetation types, and climate conditions. Additionally, our assessment provides deeper insight into the reported LST response at the intra-annual scale by distinguishing shifts in the seasonal cycle from changes in mean annual LST. For example, previous studies have reported warming across all seasons in tropical forest-loss areas.[21] Our findings attribute this phenomenon to the fact that the increase in LST trend component significantly outweighs the amplification of seasonal amplitude. In contrast, the strong seasonal variability observed in the early stages following fire is primarily driven by an amplified seasonal cycle, with its contribution surpassing that of trend components (Figures 3J and 5J). This understanding can inform a more comprehensive assessment of the ecological and societal impacts of forest loss, including its effects on crop productivity, plant phenology, species distributions, and the exacerbation of thermal stress on ecosystems.

Our results are highly consistent with temporal LST changes derived from annual and monthly LST time series (Figures S15–S17). Notably, our findings exhibit a smoother evolutionary trajectory by effectively reducing data fluctuations and minimizing commission errors caused by data loss (Figures S5–S7). Furthermore, our assessment aligns well with existing evaluations of the dynamic LST response to temporary forest loss. For example, our observations indicate that abrupt warming from boreal fires, driven by a significant reduction in LE, deviates from established latitudinal dependences of the climate feedback of forest loss.[6,7,26] This finding corroborates previous reports of such discrepancies.[11,14] We also observed a recovery dynamic in LST response to forest disturbances in mid-latitudes, which is consistent with previous studies conducted in the United States.[13] These comparisons underscore the robustness and reliability of our assessment. By incorporating a change-detection method, we specify the study period for each forest-loss pixel as extending from one year before the loss to either the end of the time series or the next abrupt change, which may be related to other disturbances.[12,56] This approach ensures that the observed LST dynamics are exclusively attributed to forest loss, thereby reducing uncertainties in biophysical diagnosis and enhancing comparability across different forest-loss events. Additionally, our method is applicable to a wide range of land-surface disturbances, enabling the identification of the dynamic processes affecting surface radiation balance.

However, it is essential to acknowledge the limitations of our assessment. While the improved BEAST is effective in detecting changes in LST, it introduces certain uncertainties. Accurately characterizing LST trajectories, particularly under disturbances, remains a significant challenge due to the use of a fixed model and parameters for global analysis.[12,34] The remainder component (i.e., residual) derived from the improved BEAST may contain valuable information related to changes in surface conditions, extreme weather events, or atmospheric circulation patterns.[57] Ignoring these residuals may introduce uncertainties into our assessments. Furthermore, the limited time span of MODIS data restricts the evaluation of the long-term impact of vegetation succession following disturbances on the local climate. For example, research on post-fire albedo trajectories indicates that increased albedo can persist for 10–35 years, with pre-fire levels typically being restored after approximately 50 years.[50,53] Consequently, our observations primarily capture progressive cooling effects in fire-affected boreal zones within this time frame. Leveraging long-term remote sensing LST data through multi-source observation fusion can enhance our understanding of the evolving biophysical climate impact of forest loss.

RESOURCE AVAILABILITY

Materials availability

This research did not generate new unique materials.

Data and code availability

The MODIS LST, albedo, and evapotranspiration can be downloaded from NASA's Level-1 and Atmosphere Archive & Distribution System (LAADS): https://ladsweb.modaps.eosdis.nasa.gov/search/. The forest-change data are available from Google Earth engine: Hansen/UMD/Google/USGS/NASA. The forest-loss-driven data are available from Science: https://www.science.org/doi/10.1126/science.aau3445. The solar incident-radiation data come from Google Earth engine: https://developers.google.com/earthengine/datasets/catalog/ECMWF_ERA5_LAND_MONTHLY. The SRTM DEM is available from Google Earth engine: https://developers.google.com/-earthengine/datasets/catalog/CGIAR_SRTM90_V4. The latest digital Köppen-Geiger world map is available from the University of Vienna: https://koeppen-geiger.vu-wien.ac.at/present.htm. The MATLAB code for processing the data is available upon reasonable request.

FUNDING AND ACKNOWLEDGMENTS

We would like to thank the three anonymous reviewers for their constructive comments. We acknowledge the support from the National Natural Science Foundation of China (grant no. 41921001) and the China Postdoctoral Science Foundation (certificate number 2023M733812). The funders had no role in study design, data collection and analysis, decision to publish, or preparation of the manuscript.

AUTHOR CONTRIBUTIONS

Conceptualization, J.L. and Z.-L.L., and Z.J.; Methodology, J.L., X.L., and Y.L.; Investigation, L.H. and M.S.; visualization, J.L., M.L., N.Y., and W.L.; supervision, H.W., R.T., and C.Z.; writing – original draft, J.L., Z.-L.L., X.L., Y.L., N.Y., and Z.J.; writing – review & editing, W.Z., S.-B.D., P.L., E.Z., and B.-H.T. All authors contributed to the manuscript and approved the final version.

DECLARATION OF INTERESTS

The authors declare no conflicts of interest.

SUPPLEMENTAL INFORMATION

It can be found online at https://doi.org/10.1016/j.xinn.2025.100875.

REFERENCES

1. Hansen, M.C., Potapov, P.V., Moore, R. et al. (2013). High-Resolution Global Maps of 21st-Century Forest Cover Change. *Science* **342**:850–853. DOI:https://doi.org/10.1126/science.1244693.
2. Food and Agriculture Organization of the United Nations (FAO) (2020). Global Forest Resources Assessment 2020 – Key Findings (FAO). DOI:https://doi.org/10.4060/ca8753en.
3. Pielke, R.A. (2005). Land Use and Climate Change. *Science* **310**:1625–1626. DOI:https://doi.org/10.1126/science.1120529.
4. Bala, G., Caldeira, K., Wickett, M. et al. (2007). Combined climate and carbon-cycle effects of large-scale deforestation. *Proc. Natl. Acad. Sci. USA* **104**:6550–6555. DOI:https://doi.org/10.1073/pnas.0608998104.
5. Bright, R.M., Davin, E., O'Halloran, T. et al. (2017). Local temperature response to land cover and management change driven by non-radiative processes. *Nat. Clim. Change* **7**:296–302. DOI:https://doi.org/10.1038/nclimate3250.
6. Bonan, G.B. (2008). Forests and Climate Change: Forcings, Feedbacks, and the Climate Benefits of Forests. *Science* **320**:1444–1449. DOI:https://doi.org/10.1126/science.1155121.
7. Lee, X., Goulden, M.L., Hollinger, D.Y. et al. (2011). Observed increase in local cooling effect of deforestation at higher latitudes. *Nature* **479**:384–387. DOI:https://doi.org/10.1038/nature10588.
8. Feddema, J.J., Oleson, K.W., Bonan, G.B. et al. (2005). The Importance of Land-Cover Change in Simulating Future Climates. *Science* **310**:1674–1678. DOI:https://doi.org/10.1126/science.1118160.
9. Seneviratne, S.I., Phipps, S.J., Pitman, A.J. et al. (2018). Land radiative management as contributor to regional-scale climate adaptation and mitigation. *Nat. Geosci.* **11**:88–96. DOI:https://doi.org/10.1038/s41561-017-0057-5.
10. Swingland, I.R., Bettelheim, E.C., Grace, J. et al. (2002). The influence of land-use change and landscape dynamics on the climate system: relevance to climate-change policy beyond the radiative effect of greenhouse gases. *Phil. Trans. Roy. Soc. Lond.* **360**:1705–1719. DOI:https://doi.org/10.1098/rsta.2002.1027.

11. Liu, Z., Ballantyne, A.P. and Cooper, L.A. (2018). Increases in Land Surface Temperature in Response to Fire in Siberian Boreal Forests and Their Attribution to Biophysical Processes. *Geophys. Res. Lett.* **45**:6485–6494. DOI:https://doi.org/10.1029/2018GL078283.
12. Li, J., Li, Z.-L., Wu, H. et al. (2022). Trend, seasonality, and abrupt change detection method for land surface temperature time-series analysis: Evaluation and improvement. *Remote Sens. Environ.* **280**:113222. DOI:https://doi.org/10.1016/j.rse.2022.113222.
13. Li, Y., Liu, Y., Bohrer, G. et al. (2022). Impacts of forest loss on local climate across the conterminous United States: Evidence from satellite time-series observations. *Sci. Total Environ.* **802**:149651. DOI:https://doi.org/10.1016/j.scitotenv.2021.149651.
14. Liu, Z., Ballantyne, A.P. and Cooper, L.A. (2019). Biophysical feedback of global forest fires on surface temperature. *Nat. Commun.* **10**:214. DOI:https://doi.org/10.1038/s41467-018-08237-z.
15. Zhang, Y., Wang, X., Lian, X. et al. (2024). Asymmetric impacts of forest gain and loss on tropical land surface temperature. *Nat. Geosci.* **17**:426–432. DOI:https://doi.org/10.1038/s41561-024-01423-3.
16. Yettella, V. and England, M.R. (2018). The Role of Internal Variability in Twenty-First-Century Projections of the Seasonal Cycle of Northern Hemisphere Surface Temperature. *JGR. Atmospheres* **123**:149–167. DOI:https://doi.org/10.1029/2018JD029066.
17. Schwartz, M.D., Ahas, R. and Aasa, A. (2006). Onset of spring starting earlier across the Northern Hemisphere. *Glob. Change Biol.* **12**:343–351. DOI:https://doi.org/10.1111/j.1365-2486.2005.01097.x.
18. Carey, C. (2009). The impacts of climate change on the annual cycles of birds. *Philos. Trans. R. Soc. B* **364**:3321–3330. DOI:https://doi.org/10.1098/rstb.2009.0182.
19. Qian, C., Fu, C. and Wu, Z. (2011). Changes in the Amplitude of the Temperature Annual Cycle in China and Their Implication for Climate Change Research. *J. Clim.* **24**:5292–5302. DOI:https://doi.org/10.1175/JCLI-D-11-00006.1.
20. Huber, J.H., Childs, M.L., Caldwell, J.M. et al. (2018). Seasonal temperature variation influences climate suitability for dengue, chikungunya, and Zika transmission. *PLoS Neglected Trop. Dis.* **12**:e0006451. DOI:https://doi.org/10.1371/journal.pntd.0006451.
21. Alkama, R. and Cescatti, A. (2016). Biophysical climate impacts of recent changes in global forest cover. *Science* **351**:600–604. DOI:https://doi.org/10.1126/science.aac8083.
22. Liu, X., Li, Z.-L., Li, Y. et al. (2023). Local temperature responses to actual land cover changes present significant latitudinal variability and asymmetry. *Sci. Bull.* **68**:2849–2861. DOI:https://doi.org/10.1016/j.scib.2023.09.046.
23. Silvério, D.V., Brando, P.M., Macedo, M.N. et al. (2015). Agricultural expansion dominates climate changes in southeastern Amazonia: the overlooked non-GHG forcing. *Environ. Res. Lett.* **10**:104015. DOI:https://doi.org/10.1088/1748-9326/10/10/104015.
24. Wang, H., Yue, C. and Luyssaert, S. (2023). Reconciling different approaches to quantifying land surface temperature impacts of afforestation using satellite observations. *Biogeosciences* **20**:75–92. DOI:https://doi.org/10.5194/bg-20-75-2023.
25. Duveiller, G., Caporaso, L., Abad-Viñas, R. et al. (2020). Local biophysical effects of land use and land cover change: towards an assessment tool for policy makers. *Land Use Policy.* **91**:104382. DOI:https://doi.org/10.1016/j.landusepol.2019.104382.
26. Li, Y., Zhao, M., Motesharrei, S. et al. (2015). Local cooling and warming effects of forests based on satellite observations. *Nat. Commun.* **6**:6603. DOI:https://doi.org/10.1038/ncomms7603.
27. Su, Y., Zhang, C., Ciais, P. et al. (2023). Asymmetric influence of forest cover gain and loss on land surface temperature. *Nat. Clim. Change* **13**:823–831. DOI:https://doi.org/10.1038/s41558-023-01757-7.
28. Stine, A.R., Huybers, P. and Fung, I.Y. (2009). Changes in the phase of the annual cycle of surface temperature. *Nature* **457**:435–440. DOI:https://doi.org/10.1038/nature07675.
29. Xing, Z., Li, Z.-L., Duan, S.-B. et al. (2021). Estimation of daily mean land surface temperature at global scale using pairs of daytime and nighttime MODIS instantaneous observations. *ISPRS J. Photogrammetry Remote Sens.* **178**:51–67. DOI:https://doi.org/10.1016/j.isprsjprs.2021.05.017.
30. Curtis, P.G., Slay, C.M., Harris, N.L. et al. (2018). Classifying drivers of global forest loss. *Science* **361**:1108–1111. DOI:https://doi.org/10.1126/science.aau3445.
31. Harper, K.L., Lamarche, C., Hartley, A. et al. (2023). A 29-year time series of annual 300 m resolution plant-functional-type maps for climate models. *Earth Syst. Sci. Data* **15**:1465–1499. DOI:https://doi.org/10.5194/essd-15-1465-2023.
32. Radwan, T.M., Blackburn, G.A., Whyatt, J.D. et al. (2021). Global land cover trajectories and transitions. *Sci. Rep.* **11**:12814. DOI:https://doi.org/10.1038/s41598-021-92256-2.
33. Wang, K., Wan, Z., Wang, P. et al. (2005). Estimation of surface long wave radiation and broadband emissivity using Moderate Resolution Imaging Spectroradiometer (MODIS) land surface temperature/emissivity products. *J. Geophys. Res.* **110**:D11109. DOI:https://doi.org/10.1029/2004JD005566.
34. Zhao, K., Wulder, M.A., Hu, T. et al. (2019). Detecting change-point, trend, and seasonality in satellite time series data to track abrupt changes and nonlinear dynamics: A Bayesian ensemble algorithm. *Remote Sens. Environ.* **232**:111181. DOI:https://doi.org/10.1016/j.rse.2019.04.034.
35. Ge, J., Huang, X., Zan, B. et al. (2023). Local surface cooling from afforestation amplified by lower aerosol pollution. *Nat. Geosci.* **16**:781–788. DOI:https://doi.org/10.1038/s41561-023-01251-x.
36. Gu, L., Meyers, T., Pallardy, S.G. et al. (2007). Influences of biomass heat and biochemical energy storages on the land surface fluxes and radiative temperature. *J. Geophys. Res.* **112**:D02107. DOI:https://doi.org/10.1029/2006JD007425.
37. Vanden, B.S., Luyssaert, S., Davin, E.L. et al. (2015). New insights in the capability of climate models to simulate the impact of LUC based on temperature decomposition of paired site observations. *J. Geophys. Res.* **120**:2015JD023095. DOI:https://doi.org/10.1002/2015JD023095.
38. Baker, J.M. and Baker, D.G. (2002). Long-Term Ground Heat Flux and Heat Storage at a Mid Latitude Site. *Clim. Change* **54**:295–303. DOI:https://doi.org/10.1023/A:1016144718218.
39. Juang, J.-Y., Katul, G., Siqueira, M. et al. (2007). Separating the effects of albedo from eco-physiological changes on surface temperature along a successional chronosequence in the southeastern United States. *Geophys. Res. Lett.* **34**:L21408. DOI:https://doi.org/10.1029/2007GL031296.
40. Li, Y., Li, Z.-L., Wu, H. et al. (2023). Biophysical impacts of earth greening can substantially mitigate regional land surface temperature warming. *Nat. Commun.* **14**:121. DOI:https://doi.org/10.1038/s41467-023-35799-4.
41. Rigden, A.J. and Li, D. (2017). Attribution of surface temperature anomalies induced by land use and land cover changes. *Geophys. Res. Lett.* **44**:6814–6822. DOI:https://doi.org/10.1002/2017GL073811.
42. Luyssaert, S., Jammet, M., Stoy, P.C. et al. (2014). Land management and land-cover change have impacts of similar magnitude on surface temperature. *Nat. Clim. Change* **4**:389–393. DOI:https://doi.org/10.1038/nclimate2196.
43. Zhang, C., Ge, Q., Dong, J. et al. (2023). Characterizing spatial, diurnal, and seasonal patterns of agricultural irrigation expansion-induced cooling in Northwest China from 2000 to 2020. *Agric. For. Meteorol.* **330**:109304. DOI:https://doi.org/10.1016/j.agrformet.2022.109304.
44. Liu, W., Dong, J., Du, G. et al. (2022). Biophysical effects of paddy rice expansion on land surface temperature in Northeastern Asia. *Agric. For. Meteorol.* **315**:108820. DOI:https://doi.org/10.1016/j.agrformet.2022.108820.
45. Zhao, L., Lee, X., Smith, R.B. et al. (2014). Strong contributions of local background climate to urban heat islands. *Nature* **511**:216–219. DOI:https://doi.org/10.1038/nature13462.
46. Winckler, J., Reick, C.H., Bright, R.M. et al. (2019). Importance of surface roughness for the local biogeophysical effects of deforestation. *JGR. Atmospheres* **124**:8605–8618. DOI:https://doi.org/10.1029/2018JD030127.
47. Delzon, S. and Loustau, D. (2005). Age-related decline in stand water use: sap flow and transpiration in a pine forest chronosequence. *Agric. For. Meteorol.* **129**:105–119. DOI:https://doi.org/10.1016/j.agrformet.2005.01.002.
48. Roberts, S., Vertessy, R. and Grayson, R. (2001). Transpiration from Eucalyptus sieberi (L. Johnson) forests of different age. *For. Ecol. Manage.* **143**:153–161. DOI:https://doi.org/10.1016/S0378-1127(00)00514-4.
49. Goetz, S.J., Mack, M.C., Gurney, K.R. et al. (2007). Ecosystem responses to recent climate change and fire disturbance at northern high latitudes: observations and model results contrasting northern Eurasia and North America. *Environ. Res. Lett.* **2**:045031. DOI:https://doi.org/10.1088/1748-9326/2/4/045031.
50. Johnstone, J.F., Chapin III, F.S., Foote, J. et al. (2004). Decadal observations of tree regeneration following fire in boreal forests. *Can. J. For. Res.* **34**:267–273. DOI:https://doi.org/10.1139/x03-183.
51. Massey, R., Rogers, B.M., Berner, L.T. et al. (2023). Forest composition change and biophysical climate feedbacks across boreal North America. *Nat. Clim. Change* **13**:1368–1375. DOI:https://doi.org/10.1038/s41558-023-01851-w.
52. Russell, R.E., Saab, V.A., Dudley, J.G. et al. (2006). Snag longevity in relation to wildfire and postfire salvage logging. *For. Ecol. Manage.* **232**:179–187. DOI:https://doi.org/10.1016/j.foreco.2006.05.068.
53. Randerson, J.T., Liu, H., Flanner, M.G. et al. (2006). The Impact of Boreal Forest Fire on Climate Warming. *Science* **314**:1130–1132. DOI:https://doi.org/10.1126/science.1132075.
54. Fu, P. and Weng, Q. (2018). Variability in annual temperature cycle in the urban areas of the United States as revealed by MODIS imagery. *ISPRS J. Photogramm. Remote Sens.* **146**:65–73. DOI:https://doi.org/10.1016/j.isprsjprs.2018.09.003.
55. Chen, J., Dai, A. and Zhang, Y. (2019). Projected Changes in Daily Variability and Seasonal Cycle of Near-Surface Air Temperature over the Globe during the Twenty-First Century. *J. Clim.* **32**:8537–8561. DOI:https://doi.org/10.1175/JCLI-D-19-0438.1.
56. Windisch, M.G., Davin, E.L. and Seneviratne, S.I. (2021). Prioritizing forestation based on biogeochemical and local biogeophysical impacts. *Nat. Clim. Change* **11**:867–871. DOI:https://doi.org/10.1038/s41558-021-01161-z.
57. Jamali, S., Jönsson, P., Eklundh, L. et al. (2015). Detecting changes in vegetation trends using time series segmentation. *Remote Sens. Environ.* **156**:182–195. DOI:https://doi.org/10.1016/j.rse.2014.09.010.

The Innovation, Volume *6*

Supplemental Information

Contrasting temporal dynamics of land surface temperature responses to different types of forest loss

Jing Li, Zhao-Liang Li, Xiangyang Liu, Yitao Li, Meng Liu, Nanshan You, Hua Wu, Lei He, Menglin Si, Ronglin Tang, Chenghu Zhou, Wei Zhao, Si-Bo Duan, Pei Leng, Wenqi Liu, Enyu Zhao, Bo-Hui Tang, and Zhenong Jin

This PDF file includes:

Supplementary Text
Supplementary Figures (Figures S1-17)
Supplementary Tables (Table S1)

Supplementary Text

Advantages of 10-day time-series data and the effectiveness of the improved BEAST method in data loss scenarios

Figure S5 shows the change detection results using the improved BEAST method at a forest loss sample site in India, where forest loss occurred in 2008 (Figure S5a). By employing high temporal resolution LST time series, the method successfully captured the abrupt LST changes caused by the land use transition from forest to cropland and revealed the distinct trend trajectories: a warming effect following loss and the cooling effect due to afforestation, as planted forests covered the loss pixel in 2018 (Figure S5b). In contrast, the annual mean time series underestimated the LST trend due to mid-year data loss (Figure S5c). Moreover, our results generalize LST trends into key features, smoothing out annual data fluctuations and enabling users to focus on the primary gradual and abrupt changes in LST. Figure S6 provides another example of forest loss driven by commodity production in the Amazon (Figure S6a). Our results captured the warming effect of crop expansion and demonstrated that trends detected using high temporal resolution data effectively avoid overestimation caused by missing data during the rainy season (Figures S6b-c). Notably, the seasonal component proved highly sensitive to forest loss. As early as 2003, when land cover within the loss pixels began transitioning (partially changing from forest to cropland), an abrupt change was detected in the seasonal component. Results from a forest loss sample driven by fire (Figure S7) further confirmed the superiority of high temporal resolution time series in characterizing abrupt and gradual LST changes compared to annual data.

Similarly, Figure S11 shows the change detection results for albedo at the three selected sample sites. The improved BEAST effectively captured the abrupt increase in albedo associated with the transition from forest to cropland at sites 1 and 2 (Figures S11a-b), despite significant data loss at these locations (Figures S11d-e). Additionally, our findings demonstrate the method’s robustness in the presence of outliers, such as those observed in 2001 and 2009 at site 3. The method successfully detected a decrease in albedo following a fire, attributed to the deposition of black carbon on soil surfaces and dead trees.

Supplementary Figures

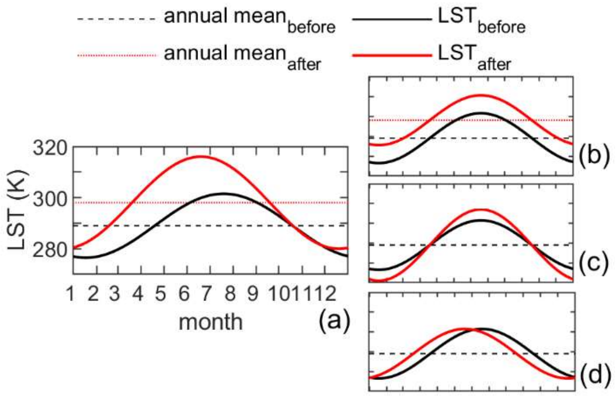

Figure S1. Framework for increase in monthly LST time series. The whole season warming (a) is driven by three distinct factors: (b) an increase in the annual mean LST, (c) an amplified amplitude of the seasonal cycle, and (d) an earlier phase shift in the seasonal cycle. Black lines represent LST time series before the change, red lines represent time series after the change. Dash or dot lines represent annual mean values.

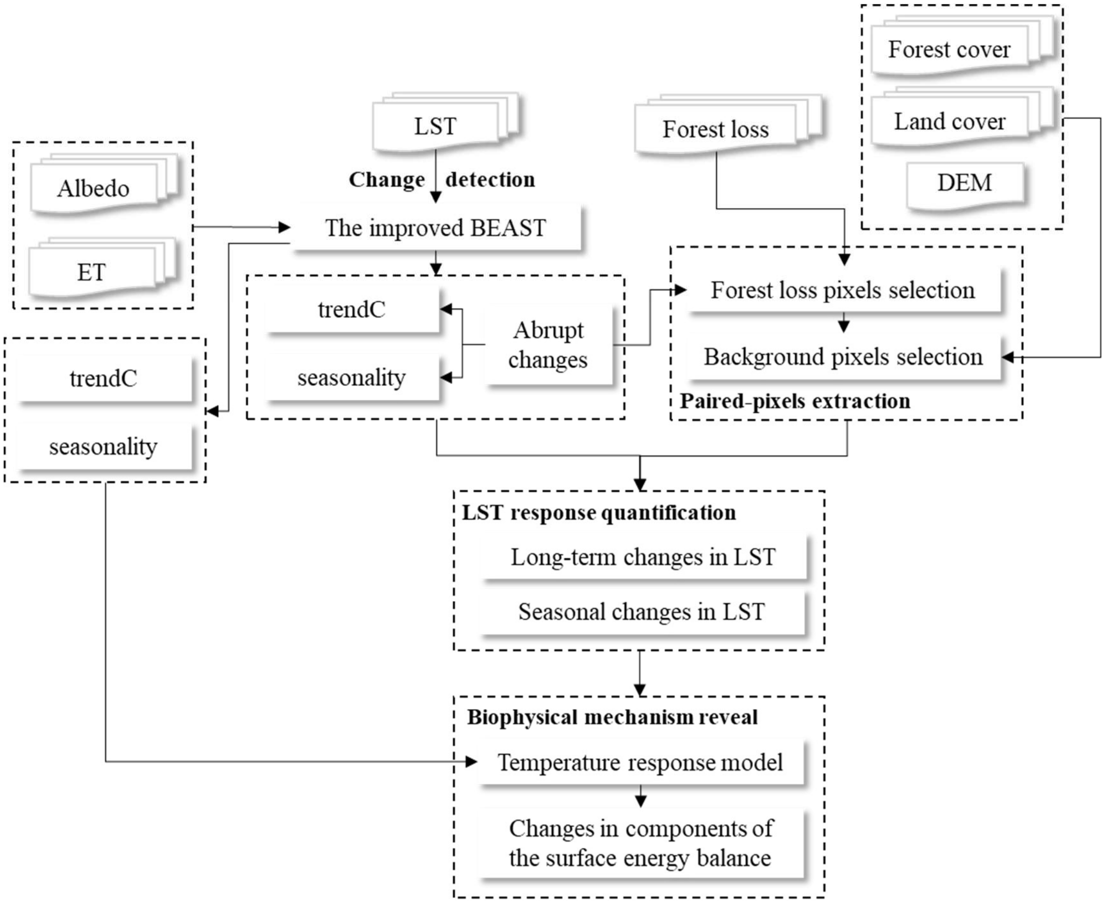

Figure S2. Flowchart of the assessment of biophysical impacts of forest loss based on satellite observations.

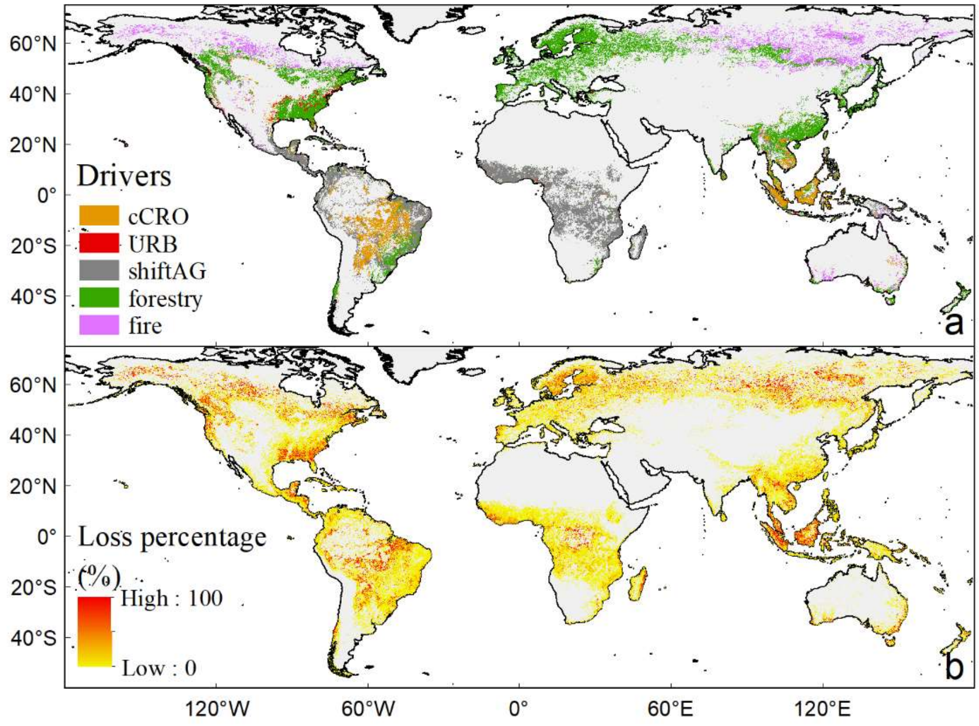

Figure S3. Spatial distribution of different forest loss types (a) and its loss percentage (b). Forest loss is primarily driven by five factors: commodity driven conversion from forest to cropland (cCRO), Urbanization (URB), shifting agriculture (shiftAG), forestry, and fire. The Grid cells with a spatial resolution of 1 km.

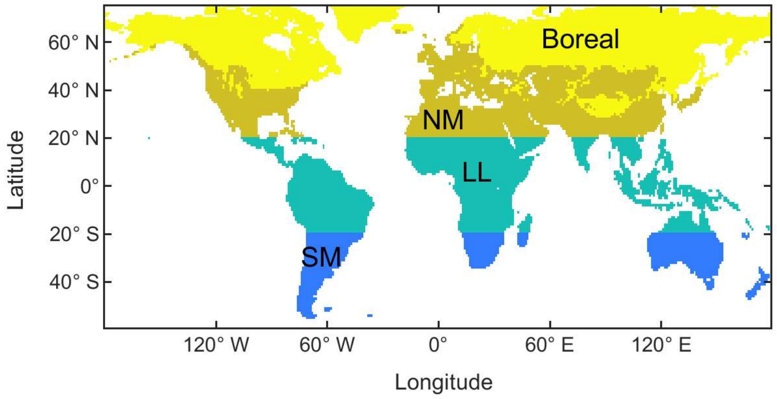

Figure S4. The distribution of different climate zones: southern mid-latitudes (SM), low latitudes (LL), northern mid-latitudes (NM), and boreal zones. The SM are the area between 60° S and 20° S, the LL are the area between 20° S and 20° N, the boreal region is the boreal zone defined on the Köppen–Geiger world map above 20° N, and the NM are the area that the left area above 20° N.

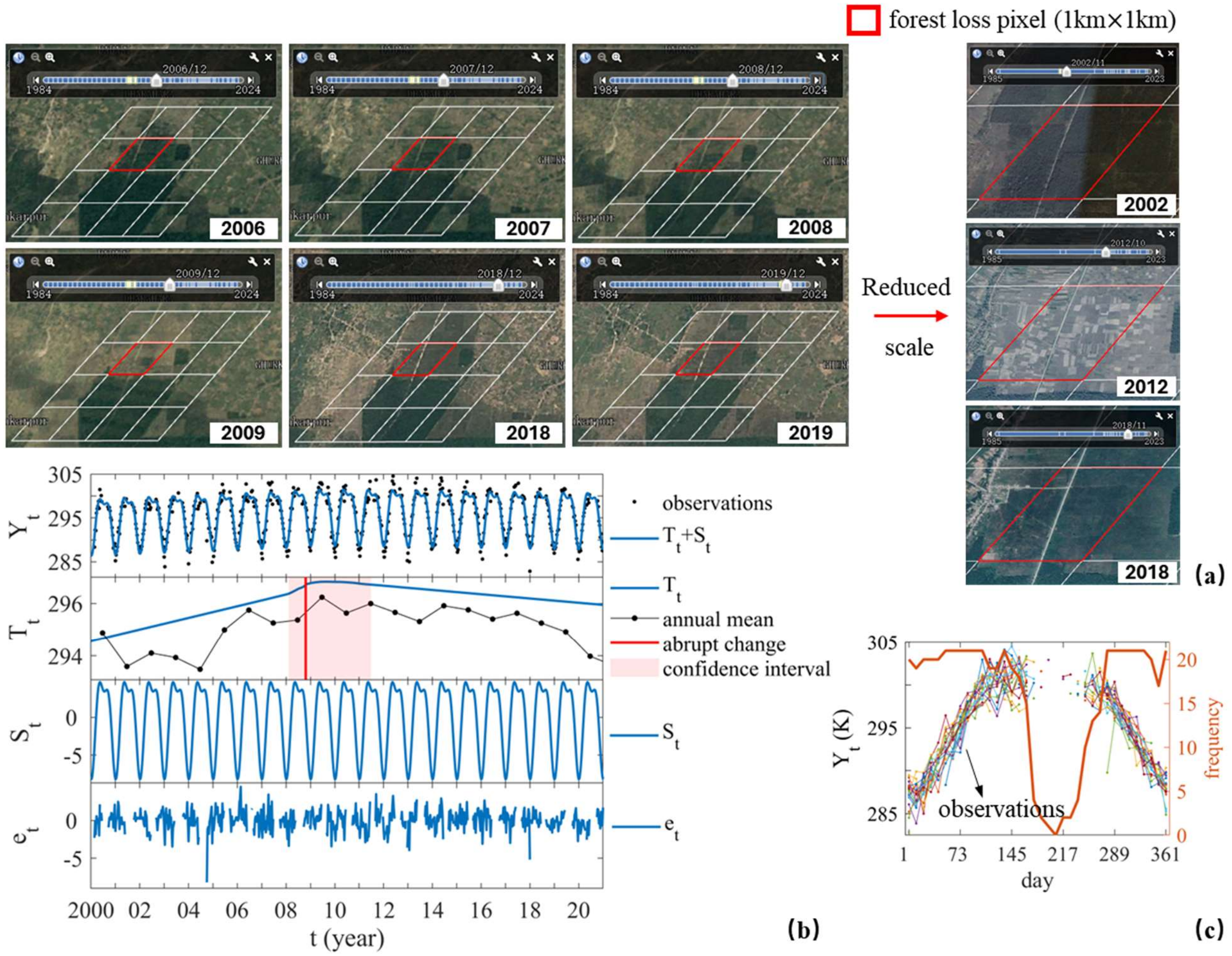

Figure S5. Forest loss sample driven by commodity production and its change detection results for LST. (a) High spatial resolution image sequences from Google Earth highlight a crop expansion after 2008 followed by afforestation in India. MODIS pixels are shown in white boxes, and the forest loss pixel is shown in the red box (central at 80.03 °E, 20.25 °N). (b) Change detection results for LST by using the improved BEAST. The LST observations with a temporal resolution of 10-day are displayed as black dots. The decomposed trend (T_t), seasonal (S_t), and remainder (e_t) components are displayed as blue lines. The annual mean LST is displayed as black line with dots, and the detected abrupt changes and their confidence intervals are shown as red lines and light red fill. (c) The observed LST time series and their frequency in the intra annual scale. The 'day' in x-axis label refers to the specific day of the year.

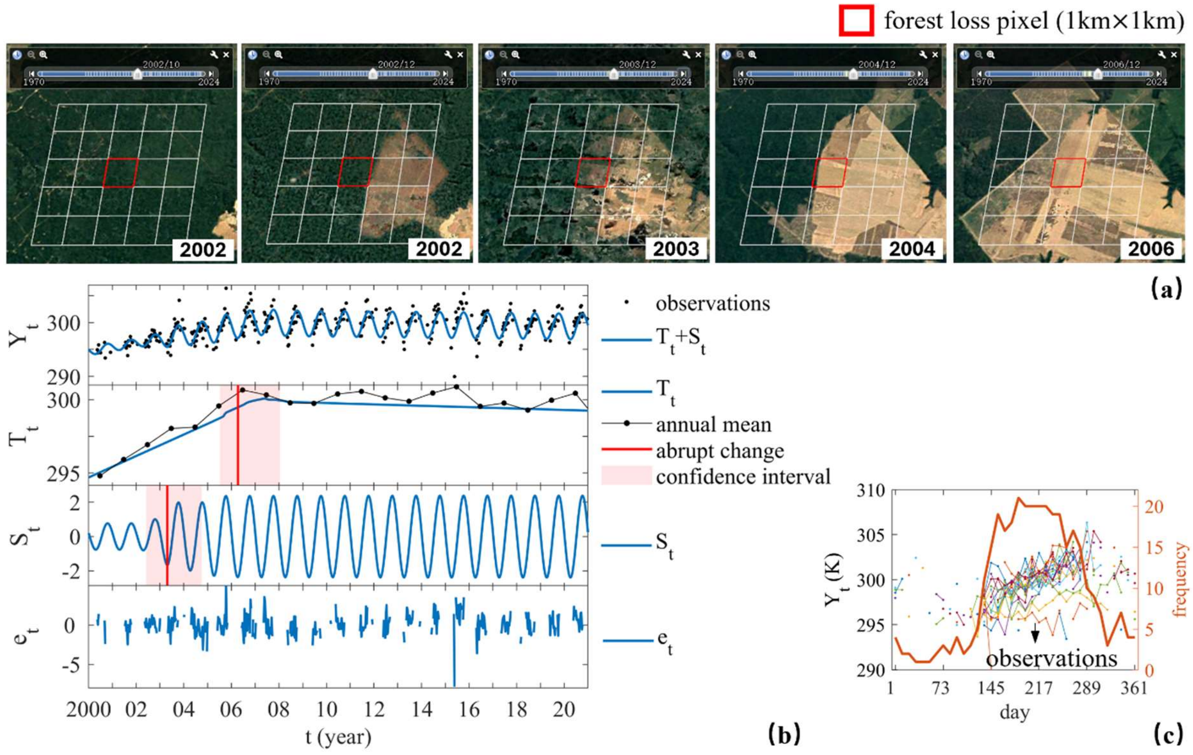

Figure S6. Same as Figure S5 but for a forest loss sample driven by commodity after 2003 in the Brazilian Amazon (central at 47.87 °W, 3.87 °S).

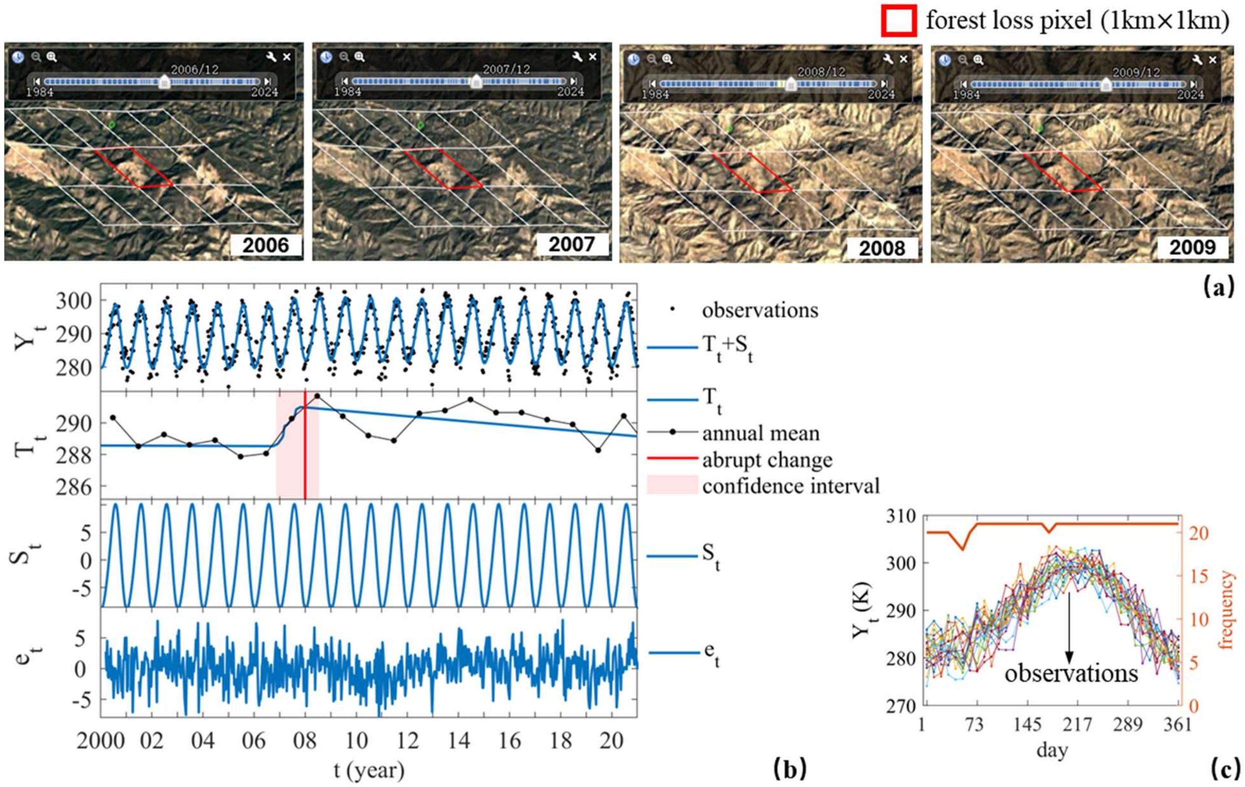

Figure S7. Same as Figure S5 but for a forest loss sample driven by wildfire that occurred in California, the USA in 2008 (central at 119.59 °W, 34.64 °N).

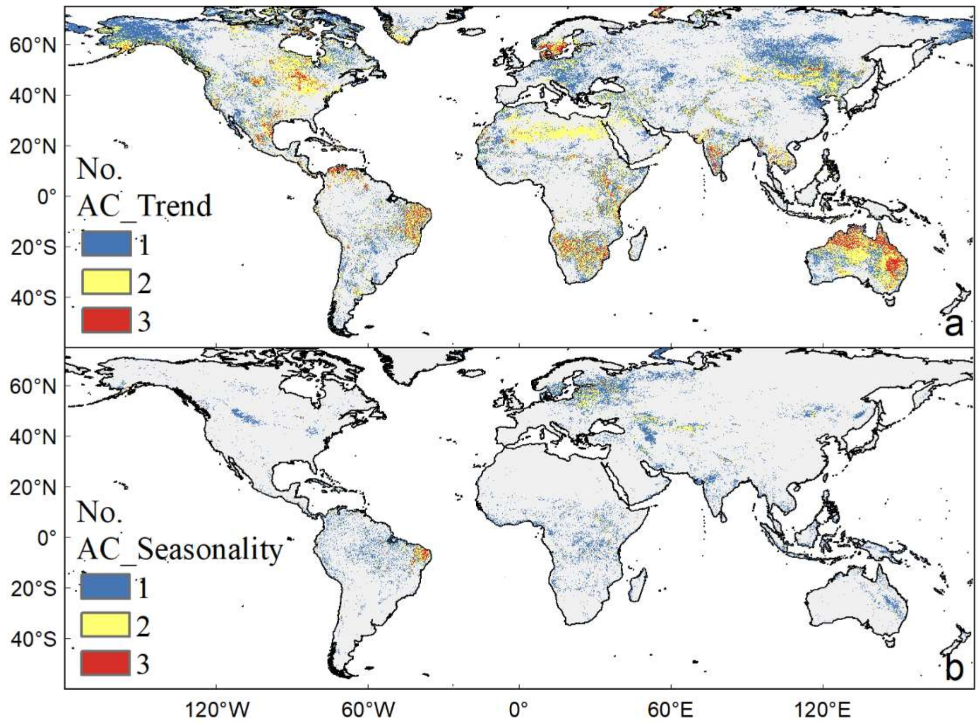

Figure S8. Abrupt changes (AC) in time-series LST. The numbers of abrupt changes in trend (a) and seasonal (b) components detected by the improved BEAST by using the MODIS LST time series. The Grid cells with a spatial resolution of 1km.

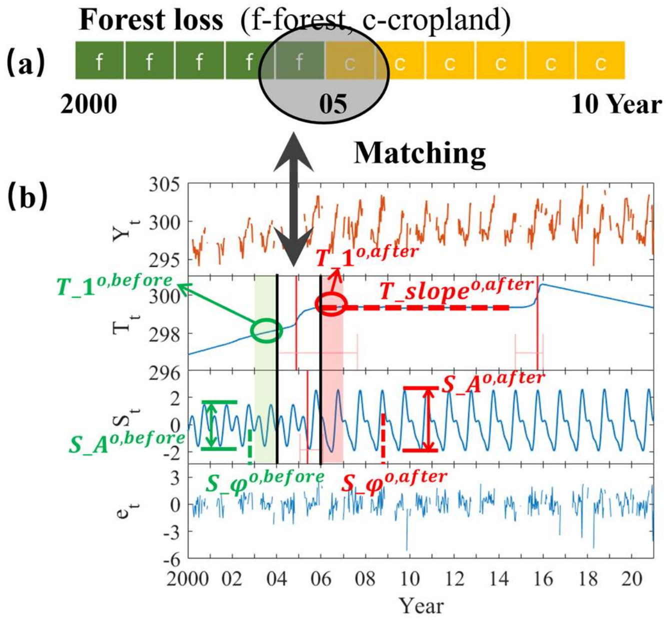

Figure S9. Illustration of the matching process between (a) a known forest loss event and (b) the corresponding detected abrupt changes in LST. (b) is the same as the Figure S10 but for commodity-driven forest loss in 2005 in the Brazilian Amazon. The calculation of the changes in LST trend and seasonality for a forest loss pixel are shown.

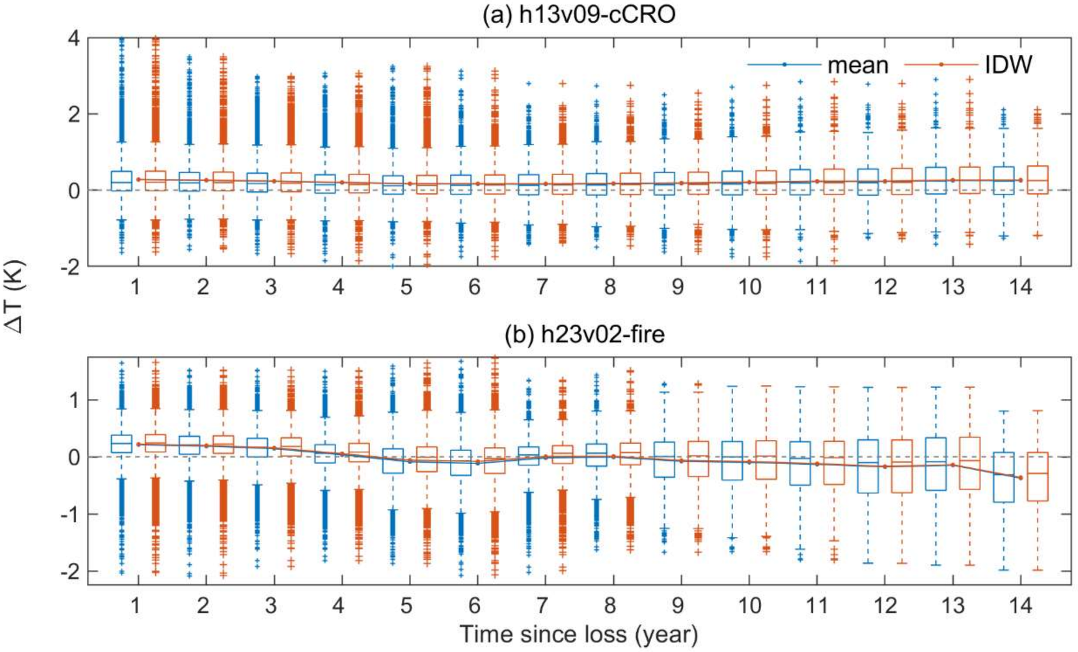

Figure S10. **Temporal dynamics of Land Surface Temperature (LST) trendC (ΔT) from 1 year to 14 years after the forest loss at two selected MODIS tiles: h13v09 and h23v02.** The primary causes of forest loss were cropland conversion (cCRO) (a) and fire (b), respectively. The ΔT dynamics was calculated by employing two different methods for extracting background pixel signals: the mean method (blue boxes) and the Inverse Distance Weighting (IDW) method (red boxes). The lines indicate the mean values of ΔT derived from the two extraction methods (mean and IDW).

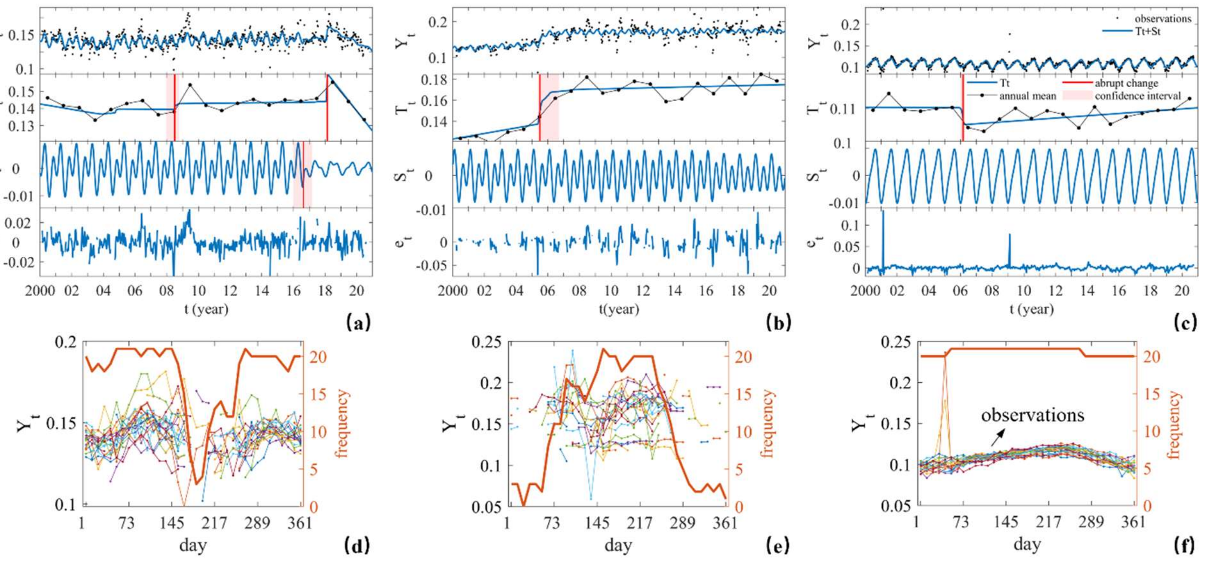

Figure S11. Same as Figure S10 but for change detection results for albedo.

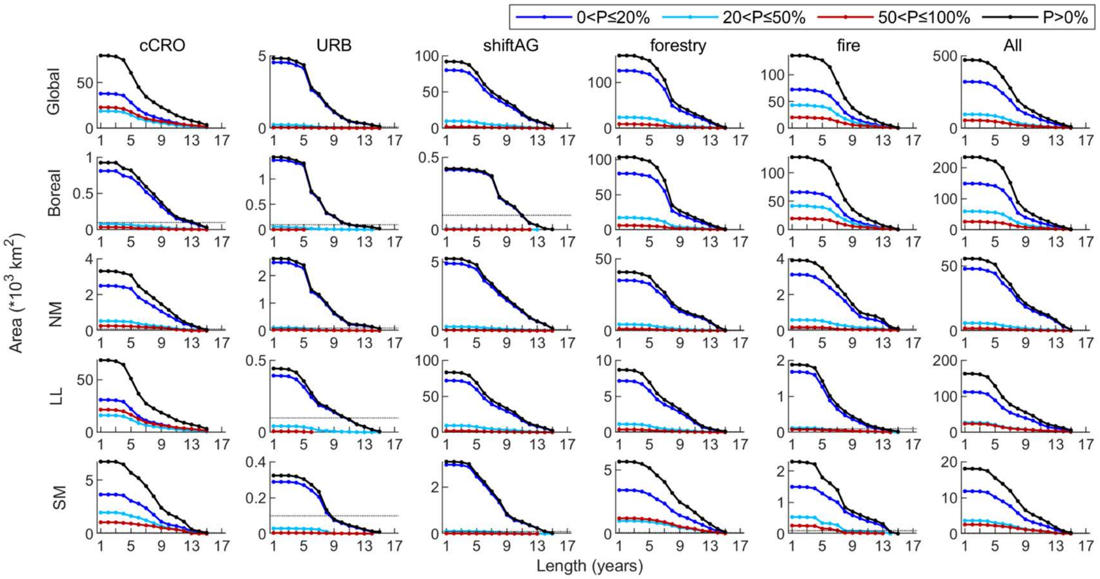

Figure S12. The area of extracted forest loss event driven by cCRO, URB, shiftAG, forestry, fire, and all types of forest loss across global, boreal, NM, LL, and SM zones. P is the percentage of forest loss.

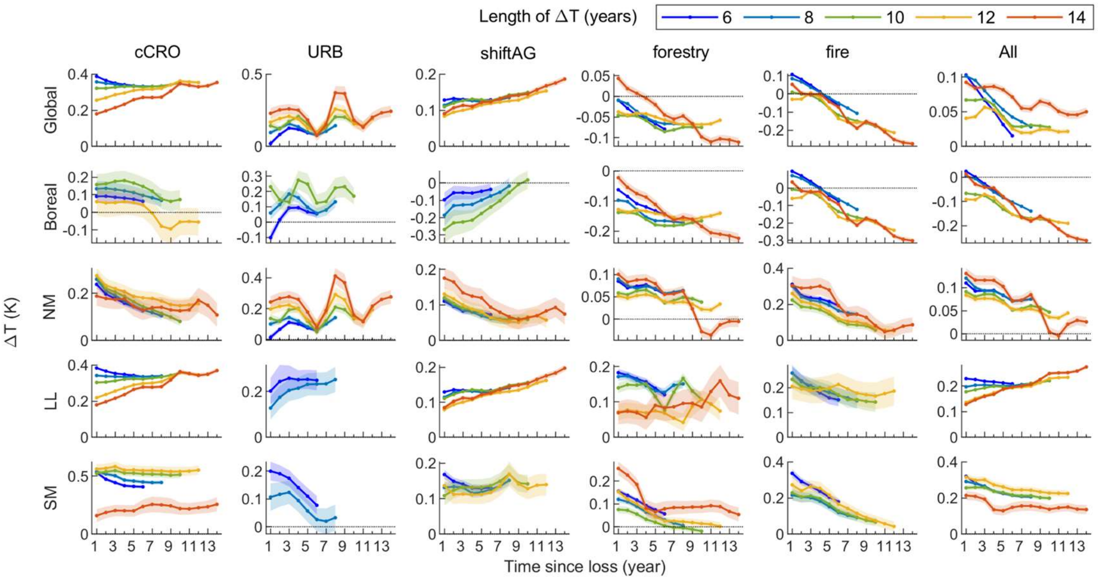

Figure S13. Temporal dynamics of the ΔT from one year to 14 years after the forest loss driven by cCRO, URB, shiftAG, forestry, fire, and all types of forest loss across global, boreal, NM, LL, and SM zones. Lines of different colors represent the different lengths (6 to 14 years) of ΔT. The filled area represents 95% confidence intervals.

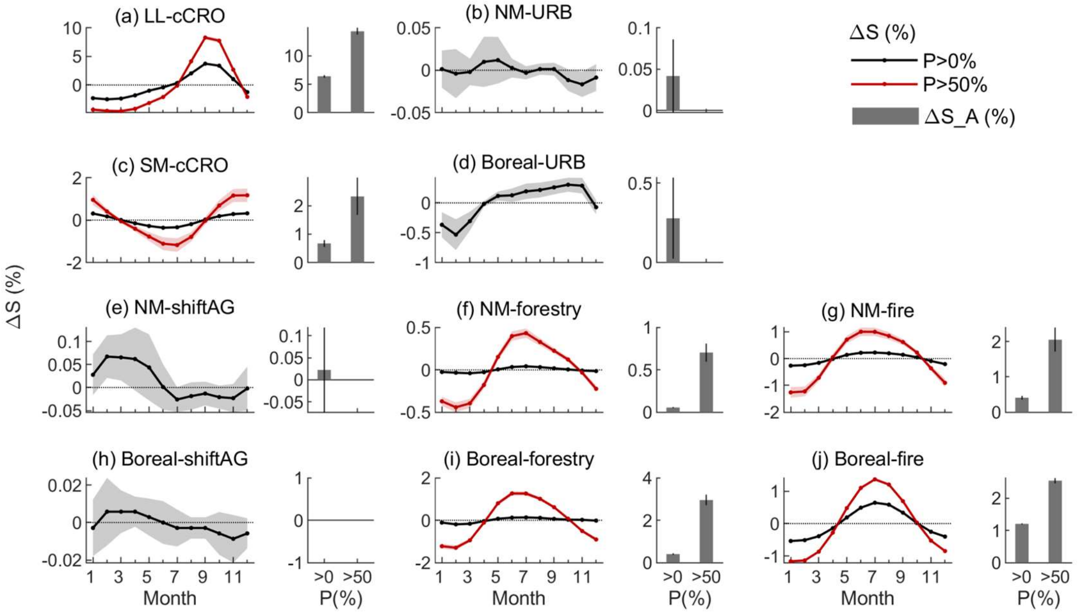

Figure S14. Relative changes in seasonal component in different months (ΔS) and seasonal amplitude (ΔS_A) caused by the forest loss driven by cCRO, URB, shiftAG, forestry, and fire across different climate zones. Subfigure titles indicate 'climate zone-forest loss type'. Colored lines on the left side represent ΔS with loss percentage (P) greater than 0% (black) and 50% (red), respectively. The filled area represents 95% confidence intervals.

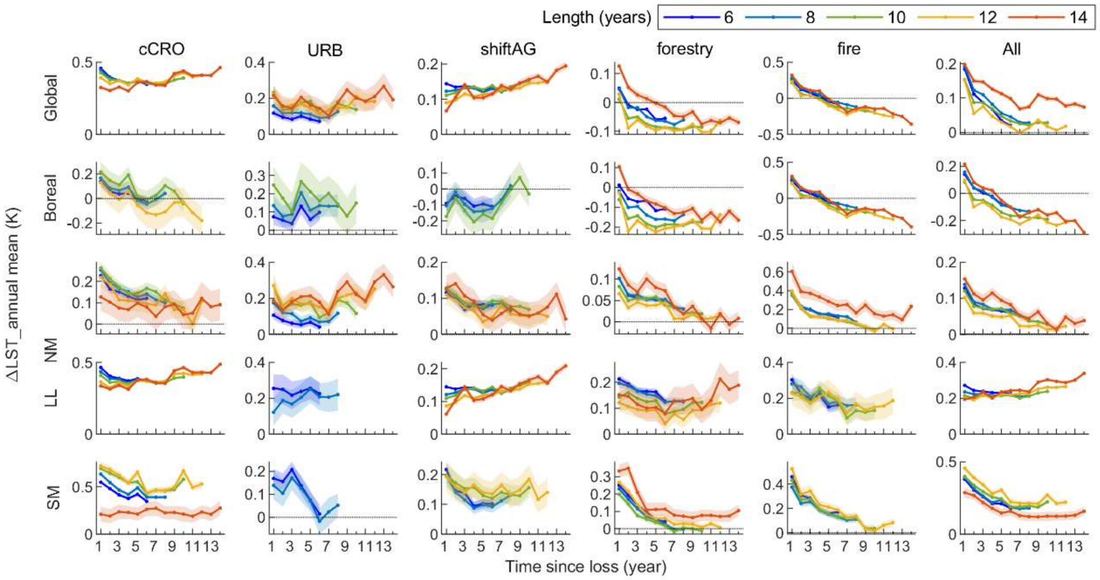

Figure S15. Temporal dynamics of the $\Delta LST\_annual\ mean$ from one year to 14 years after the forest loss driven by cCRO, URB, shiftAG, forestry, fire, and all types of forest loss across Global, Boreal, NM, LL, and SM boreal zones. Lines of different colors represent the different lengths of $\Delta LST\_annual\ mean$. The filled area represents 95% confidence intervals.

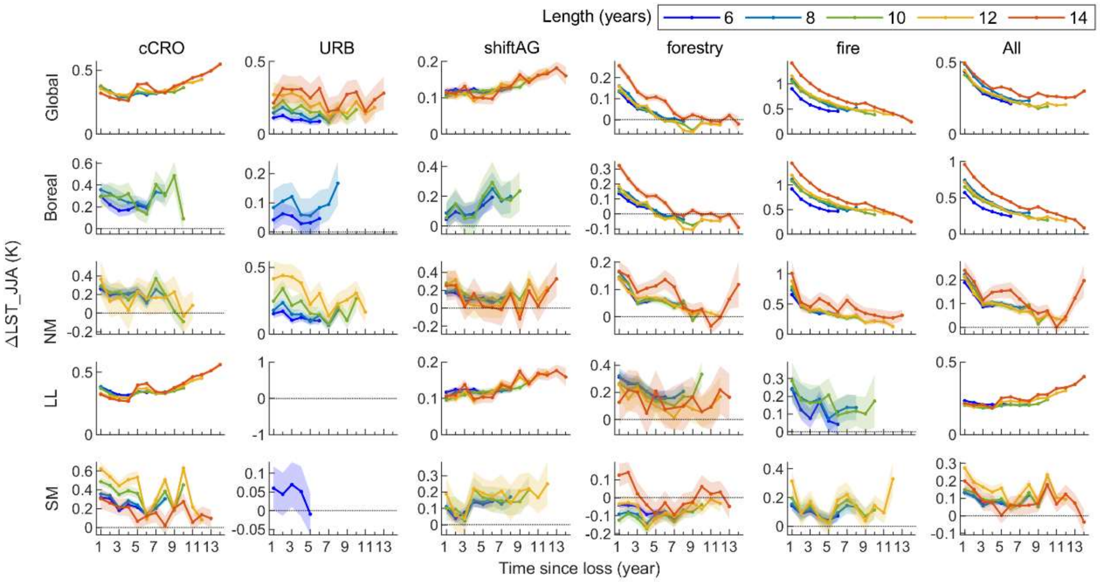

Figure S16. Same as FigureS15 but for ΔLST_JJA. JJA represents June-July-August.

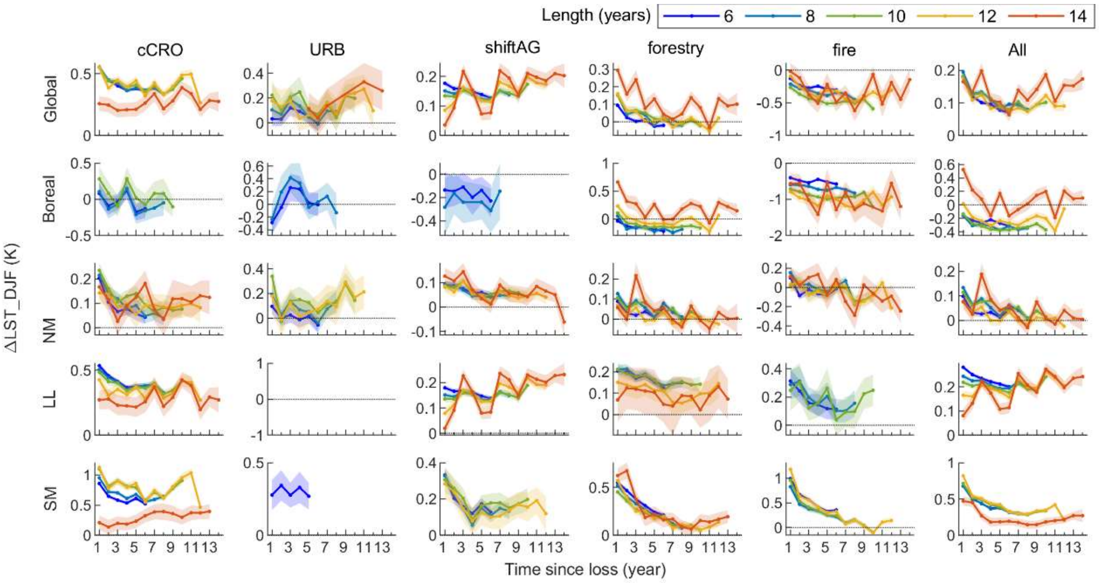

Figure S17. Same as Figure S15 but for ΔLST_DJF. DJF represents December-January-Feburary.

Supplementary Tables

Table S1. Area percentages of different change patterns of LST trendC following forest loss across different climate zones.

Abrupt impact	Long-term impact following abrupt impact	Climate zones				
		SM (%)	LL (%)	NM (%)	Boreal (%)	Global (%)
warming	enhanced warming (+ +)	0.6	6.4	1.6	2.8	11.4
	abrupt warming (+ ~)	0.9	10.1	4.8	6.1	21.9
	attenuated warming (+ -)	1.3	9.5	4.9	13.1	28.8
cooling	attenuated cooling (- +)	0.4	3.7	2.7	3.5	10.4
	abrupt cooling (- ~)	0.4	4.2	4.6	5.5	14.8
	enhanced cooling (- -)	0.3	1.9	2.1	7.9	12.2
insignificant change (~)		0.0	0.1	0.1	0.2	0.4
sum		4.0	36.0	20.8	39.1	100.0
